# Somatic mutations and single-cell transcriptomes reveal the root of malignant rhabdoid tumours

**DOI:** 10.1038/s41467-021-21675-6

**Published:** 2021-03-03

**Authors:** Lars Custers, Eleonora Khabirova, Tim H. H. Coorens, Thomas R. W. Oliver, Camilla Calandrini, Matthew D. Young, Felipe A. Vieira Braga, Peter Ellis, Lira Mamanova, Heidi Segers, Arie Maat, Marcel Kool, Eelco W. Hoving, Marry M. van den Heuvel-Eibrink, James Nicholson, Karin Straathof, Liz Hook, Ronald R. de Krijger, Claire Trayers, Kieren Allinson, Sam Behjati, Jarno Drost

**Affiliations:** 1 Princess Máxima Center for Pediatric Oncology, 3584CS Utrecht, the Netherlands; 2 Oncode Institute, 3584CS Utrecht, the Netherlands; 3 Wellcome Sanger Institute, Hinxton, Saffron Walden, CB10 1SA UK; 4 Cambridge University Hospitals NHS Foundation Trust, Cambridge, CB2 0QQ UK; 5 Department of Pathology, University of Cambridge, Cambridge, CB2 1QP UK; 6 Amsterdam UMC, University of Amsterdam, Amsterdam, the Netherlands; 7 Department of Pediatric Hemato-Oncology, University Hospital Leuven, Leuven, Belgium; 8 Hopp Children’s Cancer Center (KiTZ), 69120 Heidelberg, Germany; 9 Division of Pediatric Neurooncology, German Cancer Research Center DKFZ and German Cancer Consortium DKTK, 69120 Heidelberg, Germany; 10 Department of Paediatrics, University of Cambridge, Cambridge, CB2 0QQ UK; 11 UCL Great Ormond Street Hospital Institute of Child Health Biomedical Research Centre, London, WC1N 1EH UK; 12 Great Ormond Street Hospital for Children NHS Foundation Trust, London, WC1N 3JH UK; 13 Department of Pathology, University Medical Center Utrecht, 3584CX Utrecht, the Netherlands

**Keywords:** Cancer models, Genomic analysis, Embryonal neoplasms, Transcriptomics

## Abstract

Malignant rhabdoid tumour (MRT) is an often lethal childhood cancer that, like many paediatric tumours, is thought to arise from aberrant fetal development. The embryonic root and differentiation pathways underpinning MRT are not firmly established. Here, we study the origin of MRT by combining phylogenetic analyses and single-cell mRNA studies in patient-derived organoids. Comparison of somatic mutations shared between cancer and surrounding normal tissues places MRT in a lineage with neural crest-derived Schwann cells. Single-cell mRNA readouts of MRT differentiation, which we examine by reverting the genetic driver mutation underpinning MRT, *SMARCB1* loss, suggest that cells are blocked en route to differentiating into mesenchyme. Quantitative transcriptional predictions indicate that combined HDAC and mTOR inhibition mimic MRT differentiation, which we confirm experimentally. Our study defines the developmental block of MRT and reveals potential differentiation therapies.

## Abstract (web summary)

Malignant rhabdoid tumours (MRT) have been suggested to originate in the ectoderm-derived neural crest. Here, the authors analyse MRTs using phylogenetics, scRNA-seq, and patient-derived organoids; they find evidence for an MRT origin in the neural crest lineage and suggest differentiation treatment with HDAC/mTOR inhibitors.

## Introduction

Malignant rhabdoid tumours (MRT) are soft tissue cancers that predominantly affect infants. Although they may arise in any body part, MRT usually form in isolation or synchronously in the kidney and the brain (where they are referred to as atypical teratoid/rhabdoid tumours (AT/RT)). MRT, especially metastatic MRT, remain one of the most lethal childhood cancers, even following intense multimodal treatment. The sole driver event of MRT is the occurrence of biallelic mutations in the genes encoding *SMARCB1* (INI1, 95% of cases) or *SMARCA4* (BRG1, 5% of cases), the core subunits of the SWItch/Sucrose Non-Fermentable (SWI/SNF) chromatin-remodelling complex^1–3^. In about one-third of cases, one of the variants is present in the germline, thus predisposing children to the development of MRT^4^.

Like most childhood cancers^5^, MRT are thought to arise during embryogenesis, a notion that has recently been substantiated in studies of mouse models of *Smarcb1* loss. Rhabdoid tumours, albeit the majority being AT/RT, only developed in these mice when *Smarcb1* was inactivated during very early embryogenesis, but not at later fetal stages or in adult animals. Renal MRT were never observed^6^. Analyses of bulk, and more recently of single-cell transcriptomes, suggest that MRT retain an overall fetal transcriptome with neural as well as mesenchymal signals^7–10^. These findings suggest as a plausible source of rhabdoid tumours the ectoderm-derived neural crest, which is uniquely capable of generating cell types across the boundaries of the germ layers, mesoderm and ectoderm.

The fetal origin of MRT may be exploitable therapeutically by promoting differentiation of MRT along developmental pathways. The possibility of devising differentiation treatments for childhood cancer has recently gained traction with the advent of high-throughput single-cell assays^5,10–12^. Single-cell transcriptomic readouts enable precise, comprehensive and quantitative comparisons of cancer cells to the transcriptional changes underpinning normal cellular development, thus potentially revealing therapeutic avenues for promoting cellular maturation.

Here, we define the developmental root of MRT and reveal opportunities for differentiation therapy, combining phylogenetic analyses of tumours and surrounding normal tissues, single-cell mRNA readouts, and perturbation experiments in patient-derived MRT organoids.

## Results

### Malignant rhabdoid tumours are phylogenetically related to neural crest-derived tissues

The starting point of our investigation were phylogenetic analyses of MRT, to establish whether the origin of MRT lies in the neural crest in humans. We have previously shown that it is feasible to reconstruct the developmental relationship between childhood tumours and normal tissues from the distribution of somatic mutations across tissues^13^. Applying these principles to MRT, we used DNA whole-genome sequencing (WGS) to study two cases of MRT along with corresponding normal tissues.

**Fig. 1:**
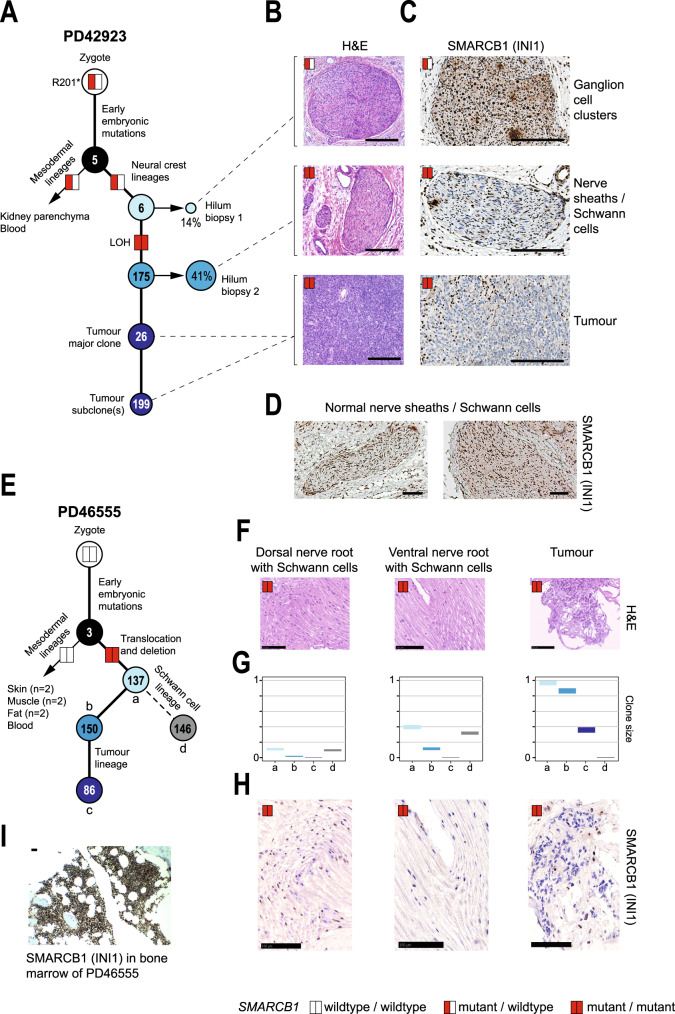
MRT are phylogenetically closely related to neural crest-derived Schwann cells. **A** Phylogenetic tree representing the somatic genetic relation of a renal MRT and normal tissues. Percentages: clone size in tissues. Numbers inside circles: mutation burden within cluster. Red or white coloured rectangles: *SMARCB1* mutations status (red = mutant; white = wild type). LOH: loss of heterozygosity. H&E (**B**) staining of biopsies and INI1 (**C**) immunostaining, showing INI1 negative Schwann cells in hilum biopsy 2. Scale bars = 100 µm. **D** Pattern of positive INI1 staining in Schwann cells of normal nerve sheath from control hilar regions of two independent donors. Scale bars = 100 µm. **E** Phylogenetic tree representing the somatic genetic relation of an extradural (spinal) MRT and normal tissues. Embryonic clusters of mutations are denoted (a–d). The annotation otherwise follows **A**. H&E staining (**F**), clone size of the different mutational clusters (a–d, **G**), and INI1 immunostaining (**H**) of dorsal nerve root, ventral nerve root, tumour and (**I**) bone marrow of the same donor. The latter showing positive INI1 staining. Scale bars = 100 µm.

First, we examined tissue obtained from a child presenting with the most common type of extracranial rhabdoid tumour, renal MRT. The child carried a pathogenic germline *SMARCB1* mutation. We performed WGS of tumour (*n* = 2), blood cells, kidney parenchyma, and renal hilar tissue (*n* = 2) (Fig. 1a, Supplementary Table 1). Using an established variant calling pipeline^13–16^, we determined somatic variants in each tissue, from which we derived phylogenetic relations between tumour and normal tissues. The possibility of observing shared mutations due to tumour contamination was addressed by histological examination and by quantitative assessment through a mixture model (“Methods” section). The key finding in this first case was that some, but not all somatic mutations of the tumour, were present in hilar tissues, occupied by ganglion cells and Schwann cells (Fig. 1a, b, Supplementary Fig. 1). Both these cell types are derived from the neural crest. However, there were no shared somatic mutations between tumour and blood or kidney parenchyma, bar ubiquitous early embryonic mutations (Fig. 1a, Supplementary Fig. 1). These findings place MRT on an ectodermal, neural crest-derived lineage with Schwann cells, distant from mesodermal blood and kidney parenchymal lineages.

Examining shared mutations between tumour and hilar tissues more closely, we found that one hilar biopsy, occupied mainly by ganglion cells, shared only a small number (*n* = 6) of variants with the tumour, whereas the second, composed of Schwann cells, shared 175 mutations with the tumour (Fig. 1a, b, Supplementary Data 1) including copy number-neutral loss of heterozygosity of *SMARCB1* (Supplementary Fig. 2). To verify this finding, we performed immunohistochemistry of the *SMARCB1* protein, INI1 (Fig. 1c). As predicted from the distribution of mutations, the first hilar biopsy showed only occasional INI1 negative cells, consistent with a heterozygous germline mutation of *SMARCB1*. By contrast, the Schwann cells of the second hilar biopsy, which should have stained ubiquitously and intensely positive for INI1 (Fig. 1d), did not exhibit INI1 staining (Fig. 1c), consistent with biallelic loss of *SMARCB1* predicted from the somatic genome of this tissue.

Next, we examined the tissues obtained post mortem from a child, who succumbed to an MRT of the cervical spine. The tumour bulk was situated ventrally in the extradural space. The child did not carry germline *SMARCB1* mutations. No early mosaic (i.e. present in blood) variant affecting *SMARCB1* was discovered in this patient. We studied tumour tissue along with nine normal tissues: skin (*n* = 2), fat (*n* = 2), muscle (*n* = 2), blood, dorsal, and ventral nerve roots (Fig. 1e, Supplementary Table 1). Pursuing the same analyses as before, we found that the tumour was somatically related to neural crest-derived Schwann cells sampled in nerve roots (Fig. 1e, f, Supplementary Fig. 1, Supplementary Data 1), but not to any other normal tissue. The clonal composition underlying this phylogenetic relation was complex. Based on variant allele frequencies and distribution of mutations, we were able to discern four clones (Fig. 1g), two of which were shared between Schwann cells and tumour. In addition, the tumour and Schwann cell lineages possessed a private clone each, alluding to a sustained potential of tumour and Schwann cells for subclonal diversification. Analysis of copy number variants (CNVs) revealed a biallelic loss of *SMARCB1* in tumour and both nerve roots (Fig. 1e, Supplementary Fig. 2, Supplementary Data 1), which again we were able to validate through INI1 staining. INI1 negative Schwann cells were more readily found in the ventral root, consistent with the larger clone sizes in this tissue (~40% vs. ~20%, Fig. 1h, i). Together, these observations provide the most direct evidence yet that human MRT is phylogenetically related to the neural crest lineage and firmly places its origin in fetal life.

### *SMARCB1* reconstitution drives MRT differentiation

**Fig. 2:**
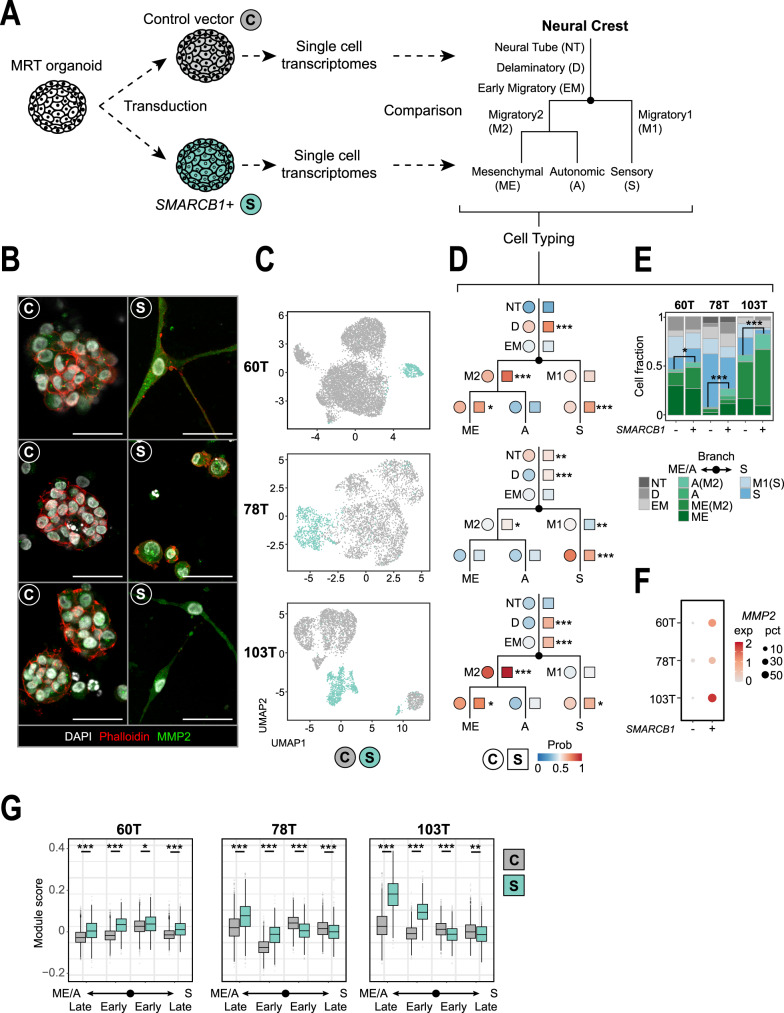
*SMARCB1* reconstitution drives MRT differentiation. **A** Schematic representation of *SMARCB1* reconstitution in patient-derived MRT organoids and subsequent single-cell transcriptome comparison to fetal mouse neural tube and neural crest cell types. Branching tree represents differentiation trajectories of mouse neural crest. Abbreviations are indicated. **B** Representative immunofluorescence images of MRT control (C) and *SMARCB1*+ (S) organoids. White: DAPI (nuclei), red: phalloidin (membranes), green: MMP2 (mesenchymal marker). Scale bars equal 50 µm. **C** UMAP representation of single cells from MRT control (grey) or *SMARCB1*+ (green) organoid lines (60T control/*SMARCB1*+: 8059/425 cells, 78T control/*SMARCB1*+: 3195/806 cells, 103T control/*SMARCB1*+: 2694/953 cells). **D** Dot plots represent similarity of MRT control (circles) or *SMARCB1*+ (squares) cells to neural crest differentiation trajectories. Colours represent the average probability (prob) that the MRT cells are similar to the indicated neural crest cell type (predicted similarity score estimated by logistic regression^12^). Changes in similarity score between control and *SMARCB1*+ cells were assessed for cell types with average similarity score >0.5. *P* values were calculated using an unpaired Student’s *t* test (two-tailed): *<1e−3, **<1e−9, ***<1e−15 (−log10 (*p* value): 60T D = 45, S = 27, M2 = 66, ME = 3.7; 78T NT = 9, D = 54, M1 = 14, S = 22, M2 = 4.4; 103T D = 198, EM = 40, S = 7.8, M2 = 314, ME = 3.2). **E** Stacked bar plot represents relative frequencies of single-cell annotations for MRT control (−) and *SMARCB1*+ (+) organoids, showing a consistent conversion of neural to mesenchymal signals. Cell type annotation was assigned for each single-cell based on the highest similarity score. Colours represent neural crest cell types depicted in Fig. 2a. Cell type migratory2 (M2) was assigned as either migratory mesenchyme (ME(M2)) or migratory autonomic (A(M2)) based on the highest similarity score. The relative frequency of the mesenchymal/autonomic (ME/A) branch was compared between control and *SMARCB1*+ organoids for each patient line. *P* values were calculated using a chi-square test: *<0.01, ***<1e−15 (*p* value: 60T = 0.0048; 78T = 4.9e−48; 103T = 1.0e−32). **F** Dot plot shows expression levels (exp) of mesenchymal marker *MMP2* for MRT control (−) and *SMARCB1*+ (+) organoids for each patient line. Colour-code from grey to red refers to average *MMP2* transcript levels (unique molecular identifier (UMI)). Dot size refers to the percentage of cells (pct) showing *MMP2* expression. **G** Box plot representation of gene module scores for MRT control (grey) and *SMARCB1*+ (green) single cells (*n* = 60T control/*SMARCB1*+: 8059/425 cells; 78T control/*SMARCB1*+: 3195/806 cells; 103T control/*SMARCB1*+: 2694/953 cells), showing consistent upregulation of mesenchymal/autonomic differentiation genes for *SMARCB1*+ cells. Box plots indicate median (middle line), 25th and 75th percentile (box). Whiskers represent the range excluding outliers (dot). Module scores were generated by averaging gene expression levels per set of genes. Gene sets include marker genes for either sensory (S) or mesenchymal/autonomic (ME/A) differentiation branches, distinguishing early and late differentiation genes. Module scores were assessed by comparing control and *SMARCB1*+ cells. *P* values were calculated using an unpaired Student’s *t* test (two-tailed): *<1e−3, **<1e−9, ***<1e−15 (−log10 (*p* value) ME/A late 60T = 28, 78T = 64, 103T = Inf; ME/A early 60T = 77, 78T = 134, 103T = Inf; S early 60T = 5.6, 78T = 72, 103T = 54; S late 60T = 28, 78T = 16, 103T = 11).

To establish the differentiation stage of MRT within neural crest development, we studied the consequences of reversing the loss of *SMARCB1*, the principal genetic driver of MRT. As a model of MRT, we utilised patient-derived MRT organoids, which have been shown to faithfully recapitulate the genetic, transcriptional, and epigenetic features of primary MRT tissue^17^. We reconstituted *SMARCB1* expression in three MRT organoid cultures^17^ (60T, 78T and 103T; Supplementary Table 2) by lentiviral transduction with either a control or *SMARCB1* expression plasmid (Fig. 2a, Supplementary Fig. 3a). DNA methylation profiles of MRT organoids resembled those of primary MRT tissue, irrespective of *SMARCB1* status (Supplementary Fig 3b). Reconstitution of *SMARCB1* expression induced a proliferation arrest in all MRT cultures (Supplementary Fig. 3c) with a morphological transformation of cells (Fig. 2b, Supplementary Fig. 4a, c). While both 60T and 103T transformed from a grape-like to a neural- or fibroblast-like morphology with long extensions protruding from the cell body, 78T stopped proliferating without an apparent morphological change. To assess the transcriptional profiles underpinning these phenotypic changes, we subjected organoid cultures, without and with *SMARCB1* re-expression, to single-cell mRNA sequencing (10× Genomics Chromium platform, *n* = 16,133 cells post filtering). Cell cycle profiles generated from single-cell transcriptomes confirmed the growth arrest induced by *SMARCB1*, with 78T showing the least penetrant effect (Supplementary Fig. 3d). UMAP clustering revealed that, as expected, most transcriptomic variance of MRT single cells can be explained by donor, as cells first separate by patient line (Supplementary Fig. 3e). After transduction, the majority of cells expressing *SMARCB1* segregated into independent cell clusters for each patient line (Fig. 2c, Supplementary Fig. 5a–c). This segregation was not explained by batch effects of individual cultures, as unsuccessfully transduced cells co-clustered with cells from cultures transduced with the control plasmid (Supplementary Fig. 3f). As our phylogenetic analyses revealed that MRT are neural crest-derived (Fig. 1), we subsequently assessed the similarity of MRT cells with and without *SMARCB1* re-expression relative to single-cell signals of (murine) neural crest development^18^ using logistic regression^12^ (Fig. 2a). At baseline (i.e. no *SMARCB1* re-expression), MRT organoid transcriptomes primarily resembled mesenchymal and neural cells (Fig. 2d, Supplementary Fig. 3g), as previously shown^7,8,10^. In addition, each individual patient line exhibited different signals of neural crest differentiation stages (Fig. 2d, Supplementary Fig. 3g). Patient lines 60T and 103T primarily resembled mesenchymal cells, whereas 78T exhibited a more neural signal. Examining cellular mRNA profiles upon *SMARCB1* reconstitution, MRT cells appeared consistently more differentiated. That is, they resembled their normal counterpart more strongly, as similarity to most neural crest cell types increased (Fig. 2d, Supplementary Fig. 3g). Further assessment of cell type similarity showed that *SMARCB1* reconstitution promotes a neural to mesenchyme conversion that is consistent among all three MRT organoid cultures (Fig. 2e). These results were validated using a second independent mouse single-cell mRNA reference of early neural and mesenchymal cell types^19^ (Supplementary Fig. 3h). In agreement, analysis of neural crest differentiation genes showed a significant upregulation of mesenchymal markers (Fig. 2b, f, g, Supplementary Fig. 4b–d, Supplementary Data 2). Additional cell typing was performed for each single-cell cluster separately to evaluate intra-organoid heterogeneity (Supplementary Fig. 5a–f, Supplementary Data 2), showing that single-cell clusters exhibited variable neural crest differentiation signals. However, *SMARCB1*+ clusters consistently induced a relative gain of mesenchymal differentiation signal, with the exception of minor cluster 60T_S2, which retained a more neural identity. Altogether, our findings place MRT on a developmental trajectory of neural crest to primarily mesenchyme conversion, which is promoted by *SMARCB1* reconstitution.

### Mimicking *SMARCB1* reconstitution pharmacologically

**Fig. 3:**
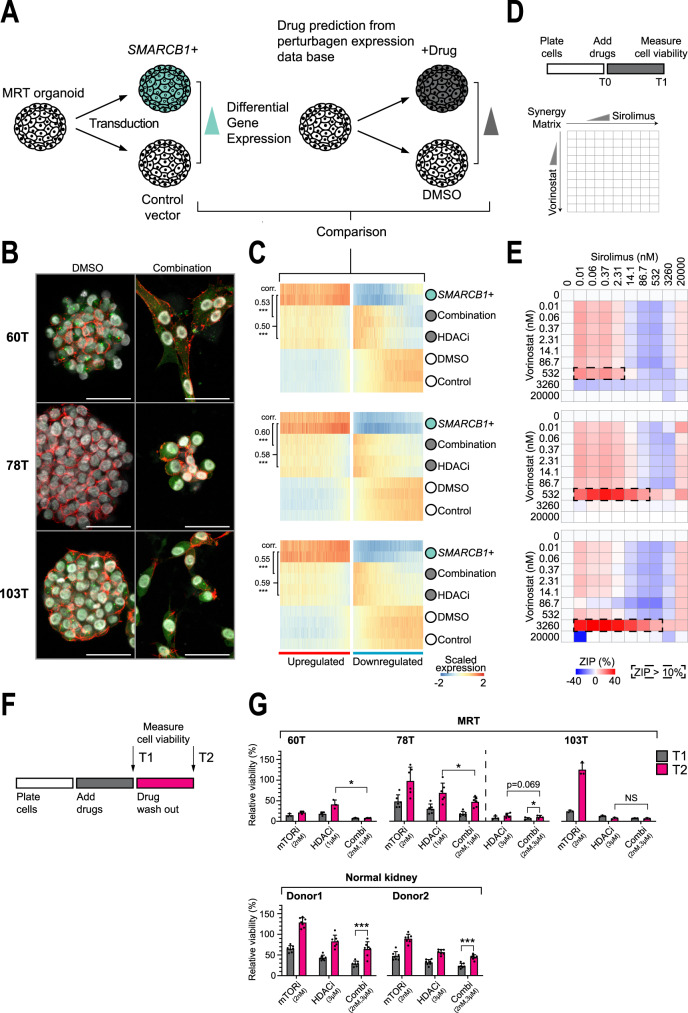
Combined HDAC/mTOR inhibition mirrors *SMARCB1* reconstitution. **A** Overview of methodology used for discovery of potential differentiation therapeutics. **B** Representative immunofluorescence images of MRT organoids treated with DMSO control or a combination of vorinostat (HDACi, 1 µM) and sirolimus (mTORi, 2 nM). White: DAPI (nuclei), red: phalloidin (membranes), green: MMP2 (mesenchymal marker). Scale bars equal 50 µm. **C** Heatmaps represent gene expression values (*n* = 2 independent experiments) of MRT control or *SMARCB1*+ organoids, or MRT organoids treated with vorinostat (HDACi, 1 µM) or both vorinostat and sirolimus (combination,1 µM/2 nM). Heatmaps are subset for genes differentially expressed upon *SMARCB1* re-expression (Supplementary Data 3). Genes are ordered by the average mRNA changes induced by *SMARCB1* re-expression and treatment. Colour-code represents gene expression values scaled by gene. Pearson correlation coefficients (corr.) were generated by comparing mRNA changes induced by either *SMARCB1* re-expression or HDACi/combination treatment. *P* values are indicated for Pearson’s correlation tests (two-tailed): ***<1e−15 (−log10 (*p* value): Combi 60T = 217, 78T = Inf, 103T = 221; HDACi 60T = 268, 78T = 306, 103T = 192). **D** Schematic overview of the dose-response matrix setup to find synergy between HDAC (vorinostat) and mTOR (sirolimus) inhibitors in MRTs. **E** Graphs show zero interaction potency (ZIP) scores that indicate either synergistic (red) or antagonistic (blue) effects of combination treatment. ZIP scores are generated by calculating the observed deviation from a reference model that assumes drugs are non-interacting (synergy when ZIP > 10%^51^). The dashed rectangles highlight the drug concentration ranges where synergy between the two drugs is the strongest. Source data are provided as a Source Data file. **F** Schematic overview of the regrowth assay. **G** Bar graphs represent cell viability values normalised to timepoint 1 (T1) DMSO controls for each MRT or normal kidney organoid line. Mean and SD (error bars) of independent experiments (dot) are indicated (*n* = 60T/103T: 3, 78T mTOR/HDAC 1 µM/Combi 2 nM 1 µM: 6, 78T HDAC 3 µM/Combi 2 nM 3 µM: 4. normal kidney: 7). Each independent experiment is an average of four technical replicates. Source data are provided as a Source Data file. Additional effect of combination treatment on cell viability was determined by comparing combination (T2) with HDACi (T2) treatment. Regrowth capability was assessed by comparing T2 to T1. *P* values were calculated using a paired ratio Student’s *t* test (two-tailed): *<0.05, **<0.01, ***<0.001 (*p* value: Combi 1 µM T1 vs. HDACi 1 µM T1 60T = 0.020, 78T = 0.012; Combi 3 µM T2 vs. Combi 3 µM T1 78T = 0.013, normal kidney donor 1 = 2.5e−5, donor 2 = 1.8e−5).

Reconstitution of *SMARCB1* to drive differentiation of MRT would appear to be an attractive, non-cytotoxic treatment strategy. However, reinstating *SMARCB1* expression genetically in children is not feasible at present. An alternative strategy is to find agents that mimic the changes induced by *SMARCB1* re-expression. Using bulk mRNA-seq, we defined a *SMARCB1*+ transcriptional programme based on genes upregulated upon *SMARCB1* re-expression in our three MRT organoid cultures (Supplementary Fig. 6a, Supplementary Data 3). We could validate the *SMARCB1*+ programme in MRT tissue and found a positive correlation with *SMARCB1* expression levels in normal tissues (Supplementary Fig. 6b). To explore therapeutic avenues, we searched a publicly available perturbation data base^20^ for drugs that induce expression changes of *SMARCB1* reconstitution (Fig. 3a). This analysis identified a variety of HDAC and mTOR inhibitors (Supplementary Fig. 6c) as the top hits. Interestingly, HDAC inhibitors have previously been identified for treatment of rhabdoid tumours by orthogonal approaches^21^. We tested the phenotypic and transcriptional effects of these agents, alone or in combination, across the three MRT organoid cultures. HDAC inhibition alone induced a morphological transformation akin to *SMARCB1* reconstitution (Fig. 3b, Supplementary Fig. 7a). Furthermore, there was a significant correlation between gene expression changes of bulk culture transcriptomes of *SMARCB1* re-expression and HDAC inhibition (Fig. 3c, Supplementary Fig. 6d). Inhibition of mTOR signalling primarily constrained organoid growth, which, however, was readily reversible by drug washout (Fig. 3f, g). Combination of HDAC and mTOR inhibition induced the phenotypic and transcriptional changes of *SMARCB1* reconstitution/HDAC inhibition as well as a marked proliferation arrest (Fig. 3b, c, Supplementary Fig. 7a). The action of HDAC and mTOR inhibition was synergistic, as corroborated by assessment of the two drugs in dose-response matrices (Fig. 3d, e, Supplementary Fig. 8a). Furthermore, the combined effects of the drugs on viability were more durable than single-agent treatment. On its own, anti-proliferation effects of each drug were readily reversible upon washout (bar HDAC inhibition in MRT organoid 103T). By contrast, combination treatment had more lasting effects on proliferation in all tested MRT organoid cultures (Fig. 3f, g, Supplementary Fig. 7b). While regrowth of 60T and 103T was completely diminished after drug washout, 78T showed minor regrowth, which could relate to the remnant proliferating cells that were also observed upon *SMARCB1* reconstitution (Supplementary Fig. 3d). To determine whether MRT are in particular sensitive to HDACi and mTOR inhibition, we tested the sensitivity of normal kidney organoids^22^ to both drugs. Normal kidney organoids were significantly more resistant to single agents as well as combination treatment, and in contrast to MRT, showed significant regrowth upon washout of drug combination (Fig. 3f, g, Supplementary Figs 7b, 8b–d). Mechanistically, the longevity of the effects of combined HDAC and mTOR inhibition may be mediated through interference with MYC, as MYC-driven cancer cell lines seem to be particularly susceptible to this drug combination^23^. In our experiments, we interrogated hallmark pathways and perturbation gene sets^24^, which validated our MRT *SMARCB1*+ model, as we found enrichment for SWI/SNF-related perturbation gene sets upon *SMARCB1* reconstitution (e.g. SNF5 (*SMARCB1*) and subunits of polycomb repressive complexes^25^) (Supplementary Fig. 6e, f). Further, we found that MYC target genes were strongly downregulated upon *SMARCB1* re-expression (Supplementary Fig. 6e, h). This was mimicked by combined HDAC/mTOR inhibition, significantly more strongly than by single-agent treatment (Supplementary Fig. 6i). Further examination of differentially expressed genes showed that identified pathways are largely shared between *SMARCB1* reconstitution and combination treatment (Supplementary Fig. 6g, Supplementary Data 3). Together, these analyses identify combined HDAC/mTOR inhibition as pharmacological mimics of *SMARCB1* reconstitution that prohibit proliferation and induce differentiation in MRT.

## Discussion

We investigated the origin of MRT by combining phylogenetic and transcriptional analyses with experiments in model systems. Our findings indicate that MRT arise from the neural crest en route to differentiating to mesenchyme and suggest combined mTOR / HDAC inhibition as a therapeutic hypothesis.

Previous investigations into the origin of MRT have built on transcriptional and epigenetic analyses of MRT. Despite the genetic uniformity of intra- (AT/RT) and extracranial rhabdoid tumours, with *SMARCB1* loss being the sole recurrent genetic driver, such analyses have revealed phenotypic, transcriptomic and epigenetic variation in rhabdoid tumours, collectively showing neural and/or mesenchymal differentiation of MRT^7,8,26,27^. Our phylogenetic analyses now firmly place the root of MRT in a lineage with neural crest-derived Schwann cells. The varied phenotype of MRT may thus be explained by the ability of neural crest lineages to generate cells across the boundaries of mesoderm and ectoderm. Modelling attempts of rhabdoid tumours in mice have shown that the timing of *Smarcb1* loss during development is critical for the formation of tumours^6,28^. It is interesting to note that in our study some morphologically normal Schwann cells partly harboured the somatic genome of MRT, including homozygous loss of *SMARCB1* in some cells. This would suggest that *SMARCB1* loss on its own may not suffice to generate tumours, or to prevent normal cellular differentiation. This proposition is further corroborated by the wide spectrum of tumours, including more benign entities, such as Schwannomas, that pathogenic germline mutations in *SMARCB1* (and related genes) predispose to^29^. Taken together, therefore, factors other than embryological timing of disruption of *SMARCB1* would seem to influence tumour formation in humans.

A unique feature that distinguishes childhood from adult cancers is the fetal origin of paediatric tumours. The developmental programmes that underpin the aberrant differentiation of cancer cells may lend themselves as therapeutic target^5^. A precedent for this notion is the clinical use of retinoic acid derivatives as maturation treatment of neuroblastoma (https://clinicaltrials.gov/ct2/show/NCT01704716). It is, however, challenging to devise maturation treatments, as conventional readouts employed in high-throughput genetic or drug screens, such as viability or proliferation, may not necessarily capture differentiation states. We employed a novel strategy here, by defining, in quantitative molecular terms, the target state of MRT differentiation, as defined through *SMARCB1* reconstitution. We then used in silico matching of target state and drug effects to search for agents that mimic genetic *SMARCB1* reconstitution. Our approach represents a specific (biased) search for agents that elicit a predefined transcriptional effect (in our case MRT maturation). Therefore, although it lacks the power of unbiased drug screens to discovering the unknown, our approach can be a focused path to drug discovery, when the target state can be defined in quantitative, molecular terms.

Studies of rare tumours, such as MRT, that rely on access to fresh material and detailed sampling are invariably small, even when conducted across large consortia, such as ours. Therefore, as the size of the biobank of MRT organoids grows with time, we will have to re-examine our observations in larger cohorts. Moreover, it will be important to examine the generalisability of our findings to other tumours driven by the biallelic loss of *SMARCB1* and other members of the BAF chromatin-remodelling complex, in particular AT/RT which are considered to be the intracranial counterpart of MRT.

MRT remains one of the most aggressive childhood cancers, which often rapidly progresses despite intense cytotoxic treatment. It would therefore seem attractive to immediately try combined mTOR / HDAC inhibition in the treatment of MRT. However, what may seem to be a harmless differentiation agent in vitro, may have severe adverse effects on postnatal development in children^5^. For instance, a phase II trial on using a sonic hedgehog (SHH) inhibitor in children with SHH medulloblastoma was terminated because of the induction of widespread growth plate fusions^30^. Nevertheless, we would suggest that the therapeutic hypothesis our findings formulate, merits further evaluation. More broadly, our study defines a nimble blueprint for quantitative approaches to the discovery of maturation targets which is applicable across childhood cancer.

## Methods

### Ethics statement

Tumour and normal tissues used for genetic lineage tracing (Supplementary Table 1) were obtained as part of the SIOP2001 study approved by the medical ethical committees of the institutes involved (Ethical Committee University Leuven (Belgium), Medical ethical committee of the Erasmus Medical Centre Rotterdam (the Netherlands)), or were obtained from the Children’s Cancer and Leukaemia Group (UK) Tissue Bank. Informed written consent was provided by all patients and/or guardians.

### Whole-genome sequencing

DNA was extracted from either formalin-fixed paraffin-embedded (FFPE) tissues or fresh frozen tumour or tissue samples. Short insert (500 bp) genomic libraries were constructed and 75 base pair (FFPE) or 150 base pair (frozen) paired-end sequencing clusters were generated on either the Illumina HiSeq X or the Illumina NovaSeq platform according to Illumina no-PCR library protocols. An overview of samples and sequencing variables, including the average sequence coverage, is shown in Supplementary Table 1.

### DNA sequence processing and mutation calling

DNA sequences were aligned to the GRCh37d5 reference genome using the Burrows–Wheeler algorithm (BWA-MEM)^31^. Single-nucleotide variants (SNVs) were called against the reference genome using CaVEMan^32^. Beyond the standard post-processing filters of CaVEMan, we removed variants affected mapping artefacts associated with BWA-MEM by setting the median alignment score of reads supporting a mutation as greater than or equal to 140 (ASMD ≥ 140) and requiring that fewer than half of the reads were clipped (CLPM = 0).

Across all samples from one patient, we recounted the SNVs that were called in any sample from the patient, using minimum values for read mapping quality (30) and base quality (25). Germline and somatic variants were distinguished using a one-sided binomial exact test on the number of variant reads and depth present in the matched tissue samples. Resulting *p* values were corrected for multiple testing with the Benjamini–Hochberg method^33^ and a cut-off was set at *q* < 10^−5^. Variants were also filtered out if they were called in a region of consistently low (<15×) or high depth (>50×) across all samples from one patient. These thresholds were halved for the X and Y chromosomes in the male patient, PD42923. Using a beta-binomial model of a site-specific error rate as previously employed^16^, we distinguished the true presence of SNVs from support due to sequencing errors and noise. All shared SNVs were further visually inspected using the genome browser, Jbrowse^34^. See Supplementary Data 1 for SNV calls of PD42923 and PD46555, respectively. CNVs were called using ASCAT^35^ and Battenberg^36^. Structural variants were called using BRASS^37^ (Supplementary Data 1).

### Clustering and classification of SNVs

To reconstruct the clonal composition of normal tissues, we employed an N-dimensional Bayesian mixture model based on the Dirichlet process^38^. Briefly, SNVs were clustered based on their distribution of variant supporting reads and total depth across all (N) samples. Therefore, it naturally accounts for differences in coverage between samples and does not rely on hard VAF cut-offs. Cellular contributions of mutational clusters were reconciled into a tree based on the pigeon hole principle, i.e. the sum of contributions in daughter branches can never exceed that of the parental one.

### Organoid culture

MRT organoids were previously established and characterised^17^. In brief, organoid line 60T, 78T and 103T (Supplementary Table 2) were seeded in growth factor-reduced BME (Trevigen) and cultured in organoid medium (Advanced DMEM/F12 (Gibco) containing 1× Glutamax, 10 mM HEPES and antibiotics (AdDF+++), supplemented with 1.5% B27 supplement (Gibco), 10% R-spondin-conditioned medium, EGF (50 ng/ml, Peprotech), FGF-10 (100 ng/ml, Peprotech), N-acetylcysteine (1.25 mM, Sigma), Rho-kinase inhibitor Y-27632 (10 µM, Abmole) and A83-01 (5 µM, Tocris Bioscience)) as described in ref. ^17^. For *SMARCB1* re-expression, MRT organoids were lentivirally transduced^39^ with pLKO.1-UbC-luciferase-blast^40^ or pLKO.1-UbC-hSMARCB1-blast lentiviruses. After 2 days, 10 µg/ml blasticidin was added to the culture medium. For DNA methylation profiling, immunofluorescence and mRNA sequencing experiments (bulk as well as single-cell), organoids were harvested 4 days after lentiviral infection. For cell viability measurements, organoids were harvested 7 days after lentiviral infection and viability was measured using CellTiter-Glo 3D reagent (Promega) according to manufacturer’s instructions on a Fluorstar Omega plate reader. HDACi in MRT organoids was performed using 1 µM entinostat (SelleckChemicals) or 1 µM vorinostat (MedChemExpress), added 2 days after seeding single cells. For HDACi and mTORi immunofluorescence and mRNA-seq experiments, 1 µM vorinostat and/or 2 nM sirolimus (MedChemExpress) were added to the organoid cultures 2 days after seeding single cells. Cells were harvested 4 days after addition of the drugs.

### Histology

Tissues were fixed in 4% paraformaldehyde, dehydrated and embedded in paraffin. Immunohistochemistry was performed according to standard protocols on 3–4 µm sections. Sections were subjected to H&E and immunohistochemical staining with the following primary antibodies: INI1 (BD Transduction Laboratories, 612111, 1:400).

### Immunofluorescence

Immunofluorescence experiments were performed as described in ref. ^41^, using DAPI (Thermo Fisher, D9542, 1:1000), Alexa Fluor 647 Phalloidin (Thermo Fisher, A22287, 1:200) and primary antibody MMP2 (Thermo Fisher, MA5-13590, 1:500). High-resolution 3D imaging was performed in µ-Slide 8 Well chambers (IBIDI, 80826) using the Leica SP8 confocal microscope and a 20× water immersion objective. Organoids were imaged in 3D by acquiring z-stacks, which were visualised by maximum intensity projections. Acquisition settings for MMP2 were fixed across experiments.

### DNA methylation profiling

Genomic DNA was extracted from MRT organoids using the ReliaPrep gDNA Tissue Miniprep System (Promega) according to the manufacturer’s protocol. DNA methylation profiles were established using Illumina Human MethylationEPIC BeadChip arrays at the German Cancer Research Center (DKFZ) Genomics and Proteomics Core Facility according to the manufacturer’s protocol. DNA methylation data were analysed as described in ref. ^26^. Organoid methylation data were co-clustered with previously published DNA methylation data of AT/RT and MRT^7^.

### Single-cell mRNA sequencing

MRT organoids were dissociated into single-cell suspensions using TrypLE Express (Thermo Fisher) supplemented with Y-27632 (10 µM, Abmole) and subsequently processed on the Chromium 10X Genomics controller as previously described^10^. Cell viability was estimated using Trypan Blue solution (Thermo Fisher, 15250061), ranging from 38 to 65% viable cells. For 78T and 103T samples, single cells were mixed pre-loading in a one to one ratio. Libraries were produced according to the manufacturer’s protocol and sequenced on Illumina platforms (HiSeq4000 or NextSeq500).

### Mapping, quantification, quality control and demultiplexing of single-cell mRNA-seq data

Raw sequencing data were aligned to the reference genome (GRCh38) and quantified with cell Ranger 2.0.2 pipeline^42^ for 60T samples (sequencing was done with Chromium single-cell 3′ v2 chemistry) and cell Ranger 3.0.3 pipeline for mixed 78T and 103T samples (sequencing was done with Chromium single-cell 3′ v3 chemistry), producing a table of counts of unique molecular identifiers (UMI) for each single-cell and gene with sequencing depth (average reads/cell) for 60T control (35k), *SMARCB1*+ (490k), 78T and 103T mixed control (34k) and *SMARCB1*+ (44k). Demultiplexing of 78T and 103T was performed with demuxlet software^43^ with default parameters using genotype data of patient line 78T and 103T^17^. Gene expression matrices were further processed with python package scanpy version 1.4.4.post1^44^. Poor quality cells were filtered out based on a low number of genes (<200 detected genes) and high mitochondrial content (>10% for 60T and >20% for 78T and 103T). Cells were filtered based on *SMARCB1* transcript counts. For MRT control samples, cells were removed with *SMARCB1* > 0. For MRT *SMARCB1*+ samples, cells were removed with *SMARCB1* = 0. Doublets were detected and excluded with python package scrublet version 0.2.1^45^ with default parameters. Demultiplexing of organoid lines 78T and 103T was performed based on cluster assignment provided by demuxlet. In addition, single-cell outliers for 78T and 103T were removed based on low coverage (detected UMIs <1500) or high counts (more than 60,000 for 78T and 50,000 for 103T). All the scripts used for filtering and quality control are listed in the “Code availability” section. Sample metadata are provided in Supplementary Table 3.

### Clustering and module scores

For UMAP visualisation and single-cell clustering, raw gene expression matrices of filtered cells were processed using R package Seurat version 3.1.4 and standard analysis pipelines^46^. Downstream analysis was performed for all single cells combined (Fig. 2f, g, Supplementary Fig. 3d, e) or separately for each patient line (Fig. 2c, Supplementary Figs. 4b, 5a–f) and included the following steps. Data normalisation of raw counts was performed using R package sctransform^47^ version 0.2.1 integrated in Seurat. The single command “SCTransform” was applied, which replaces the Seurat commands “NormalizeData”, “ScaleData” and “FindVariableFeatures”. Counts were normalised using the standard normalisation method “LogNormalize” with scaling factor 10,000. Confounding sources of variation were removed by regressing out mitochondrial content (“PercentageFeatureSet” for gene names starting with “MT”). Single cells were scored for cell cycle (“CellCycleScoring”) which was additionally regressed out to correct for unwanted cell cycle effects. Subsequently, we performed principal component analysis (“RunPCA”) using highly variable features (3000 genes using default parameters), followed by dimensionality reduction (“RunUMAP”). Inclusion of principal components was based on a scree plot (“ElbowPlot”) by placing the cut-off at the elbow of the curve (Combined: 1-18, 60T: 1–15, 78T: 1–17, 103T: 1–15). Clusters were determined by Louvain graph-based clustering, performed separately for each patient line (“FindNeighbours” and “FindClusters”). The optimal clustering resolution was identified by calculation of average silhouette widths for a series of resolutions (Supplementary Fig. 5b). Differentially expressed genes for control and *SMARCB1*+ single cells were determined using “FindMarkers” (Supplementary Data 2). Marker genes defining single-cell clusters were determined using “FindAllMarkers” (Supplementary Data 2). *P* values were calculated using a Wilcoxon Rank Sum test and were corrected for multiple testing. Module scores were calculated (“AddModuleScore”) on a combined normalised dataset of all patient lines for inter-organoid comparisons (Fig. 2g), or separately for each patient line for assessment of single-cell clusters (Supplementary Fig. 5f). Neural crest branch-specific gene sets include marker genes that separate sensory and autonomic/mesenchymal branches, further specifying late genes (upregulated in differentiated cells) and early genes (upregulated in progenitor and differentiated cells), as described in ref. ^18^. Mouse genes without human orthologue were excluded. All the scripts used for clustering and module scores are listed in the “Code availability” section.

### Logistic regression

The probability that the transcriptome for each single MRT cell is similar to each mouse neural crest cluster was estimated with logistic regression in R, as described previously^12^. The fetal mouse neural crest dataset^18^ was extracted from GEO (accession number GSE129114) selecting for the following cells: Wnt1 E8.5 whole embryo, Wnt1 E9.5 trunk and Wnt1 E10.5 tail. Cluster assignment for each cell in the combined dataset was extracted from Supplementary Table 9^18^. The second dataset of fetal mouse organogenesis^19^ was extracted from GEO (accession number GSE119945), selecting cells from early mesenchymal, glial and neural trajectories with the following cell annotations (Main_cell_type field in GSE119945_cell_annotate.csv.gz file): Early mesenchyme, Intermediate Mesoderm, Connective tissue progenitors, Schwann cell precursor, Sensory neurons, Neural Tube, Neural progenitor cells, Radial glia. Mouse gene symbols were converted to their human orthologues using biomaRt version 2.40.4 in R. Raw transcript counts for human genes that mapped to multiple mouse genes were excluded from further analysis. The logistic regression model was trained on the neural crest dataset with R package cv.glmnet, and was used to generate probability scores for the MRT cells.

### Bulk mRNA sequencing

Total RNA was extracted using Trizol reagent (Thermo Fisher) and quality-checked using Bioanalyzer2100 RNA Nano 6000 chips (Agilent). Sequencing libraries were prepared using the NEBNext^®^ Ultra™ RNA Library Prep Kit (New England Biolabs) according to the manufacturer’s protocol. Stranded paired-end sequencing (PE150) was performed on the Illumina HiSeq or Illumina NovaSeq platform by Novogene (Hong Kong).

For data analysis, TruSeq3 adapters were removed from reads using Trimmomatic version 0.36.5, followed by sliding window trimming to trim low-quality bases (<20 average quality over four bases). Unpaired reads were removed for subsequent steps. Reads were mapped to the reference genome (GRCh38) using STAR version 2.6.0 and assigned using featureCounts version 1.6.3 based on gene annotation GENCODE version 28. Gene expression changes were calculated using the R package DESeq2 version 1.22.1, and differentially expressed genes were determined by the Wald significance test with multiple-testing correction (FDR < 0.01 and fold change > 2) (Supplementary Data 3). The overlap of differentially expressed genes for three patient lines was tested using a multi-set hypergeometric test^48^. For *SMARCB1*+ programme validation, *z* scores were generated from gene expression data acquired from a paediatric renal tumour biobank^17^ and downloaded from the GTEx portal (https://gtexportal.org/home/datasets, GTEx_Analysis_2017-06-05_v8_RNASeQCv1.1.9_gene_median_tpm.gct.gz). For PCA, gene counts were normalised by variance stabilising transformation (VST, DESeq2), and principal components were generated using prcomp in R. For heatmaps, VST counts were corrected for batch effect using R package limma version 3.38.3. Heatmaps were generated using R package pheatmap version 1.0.12, scaling was applied by gene. Gene set enrichment analysis^49^ for hallmark and perturbation^24^ gene sets was performed using the R package clusterProfiler version 3.10.1. Genes were ranked according to *SMARCB1*-induced mRNA changes that were averaged for 60T, 78T and 103T. For the comparison of *SMARCB1* re-expression and combination treatment, unordered gene sets (generated by intersection of differentially expressed genes, Supplementary Data 3) were submitted for hallmark pathway enrichment analysis.

### Connectivity map

A connectivity map was generated by submitting the top 100 genes upregulated by *SMARCB1* re-expression in MRT organoids to the online CLUE query tool^20^. Input genes were selected based on significantly upregulated genes overlapping for 60T, 78T and 103T. Genes were ranked based on average gene expression changes. Connectivity map drugs were ranked by the median percentage of similarity.

### Western blot

Western blot on MRT organoids was performed as described in ref. ^50^ using the following antibodies:

SMARCB1/INI1 A-5 (Santa Cruz Biotechnology, sc-166165, 1:1000), beta-tubulin H235 (Santa Cruz Biotechnology, sc-9104, 1:1000).

### Drug testing and synergy assays

MRT organoids were harvested and washed in ice-cold AdDF+ ++. Organoids were subsequently filtered using a 70 µm cell strainer (Falcon) and resuspended in 5% BME in medium. Next, ~500 organoids were plated using the Multi-drop^TM^ Combi Reagent Dispenser on repellent black 384-well plates (Corning) to which medium with drugs were added using the Tecan D300e Digital Dispenser. Four technical replicates were included in each experiment. Five days after adding drugs, cell viability was measured using CellTiter-Glo 3D reagent (Promega) according to the manufacturer’s instructions. Results were normalised to the DMSO vehicle (100%). Dose-response curves were generated by nonlinear regression (curve fit) using GraphPad Prism v8.0.2 Synergy scores were generated by R package synergyfinder version 1.8.0 using the ZIP method^51^.

### Regrowth assay

MRT and normal kidney organoids were dissociated into single cells using mechanical disruption and TrypLE Express (Invitrogen, 12605036), and 5000 cells were seeded in 5 µl BME droplets in two separate flat-bottom 96-well plates (Corning). Drugs were added either one (78T), two (60T and 103T) or 3 (normal kidney donor 1 and 2) days after seeding to normalise for growth rate. Vorinostat and sirolimus concentration were selected based on the highest synergy score for each patient line. Sirolimus concentration was fixed for all experiments (2 nM), while vorinostat concentration varied (60T: 1 µM, 78T: 1 µM, 103T: 3 µM). Both concentrations of vorinostat (1 and 3 µM) were tested for 78T and normal kidney organoids. Five days after addition of the drugs, cell viability was measured in the first plate using CellTiter-Glo 3D reagent (Promega) according to the manufacturer’s instructions. In the second plate, medium was exchanged for medium without drugs. Five days after drug removal, cell viability was measured. Results were normalised to the DMSO vehicle of T1 (100%).

### Statistics and reproducibility

For Fig. 1, genetic lineage tracing experiments were performed for *n* = 2 independent donors. Images of Fig. 1b–d, **i** correspond to the regions that were sampled for genetic lineage tracing experiments, which were imaged before and after the collection of material (*n* = 2). Representative images of Fig. 1d were derived from *n* = 2 independent donors, for which at least *n* = 3 areas with nerve tissue were assessed for each donor. For Fig. 2b and Supplementary Fig. 4a, c, d, representative images were derived from *n* = 3 independent immunofluorescence experiments. The western blot image in Supplementary Fig. 3a was representative of *n* = 3 independent experiments. For Fig. 3b, representative images were derived from *n* = 2 independent immunofluorescence experiments. For Supplementary Fig. 7a, b, representative images were derived from at least *n* = 3 independent experiments.

### Reporting summary

Further information on research design is available in the Nature Research Reporting Summary linked to this article.

## Supplementary information

Supplementary Information

Description of Additional Supplementary Files

Supplementary Data 1

Supplementary Data 2

Supplementary Data 3

Reporting Summary

## Data Availability

Raw sequencing data have been deposited in the European Genome-phenome Archive (EGA, www.ebi.ac.uk/ega/). Accession numbers are EGAD00001006574 (bulk mRNA-seq;) and EGAD00001006296 (WGS and scRNA-seq;). The data are available under restricted access. Access can be granted by contacting biobank@prinsesmaximacentrum.nl (EGAD00001006574) or datasharing@sanger.uk.ac (EGAD00001006296). DNA methylation data are available under GEO (www.ncbi.nlm.nih.gov/geo/) accession number GSE161814. Processed scRNA-seq data (derived from raw sequencing data deposited under accession number EGAD00001006296) are available at https://github.com/kheleon/mrt-paper^52^. Content includes filtered_gene_bc_matrices and filtered_feature_bc_matrix folders from cellranger output. The fetal mouse neural crest dataset^18^ was extracted from GEO (www.ncbi.nlm.nih.gov/geo/, accession number GSE129114). The second dataset of fetal mouse organogenesis^19^ was extracted from GEO (www.ncbi.nlm.nih.gov/geo/, accession number GSE119945). Gene expression data of the paediatric renal tumour biobank^17^ was extracted from EGA (www.ebi.ac.uk/ega/, accession number EGAD00001005318 and EGAD00001005319). Gene expression data of normal tissues was extracted from GTEx portal (https://gtexportal.org/home/datasets, GTEx_Analysis_2017-06-05_v8_RNASeQCv1.1.9_gene_median_tpm.gct.gz). The remaining data are available within the Article, Supplementary Information, Supplementary Data and Source Data provided with this paper or are available from the authors upon request. Source data are provided with this paper.

## Source data

Source Data

## Code availability

Jupyter notebooks for processing of scRNA-seq data are available at https://github.com/kheleon/mrt-paper^52^.
