# Supplementary material for: Somatic mutations and single-cell transcriptomes reveal the root of malignant rhabdoid tumours
Source: Nat Commun. 2021 Mar 3;12:1407. doi: 10.1038/s41467-021-21675-6 (PMC7930245; doi:10.1038/s41467-021-21675-6)
Supplement: Supplementary file 1 — Supplementary Information [file 41467_2021_21675_MOESM1_ESM.pdf]

Supplementary Information

Somatic mutations and single cell transcriptomes reveal the root of malignant rhabdoid tumours

Custers, Khabirova, Coorens et al.

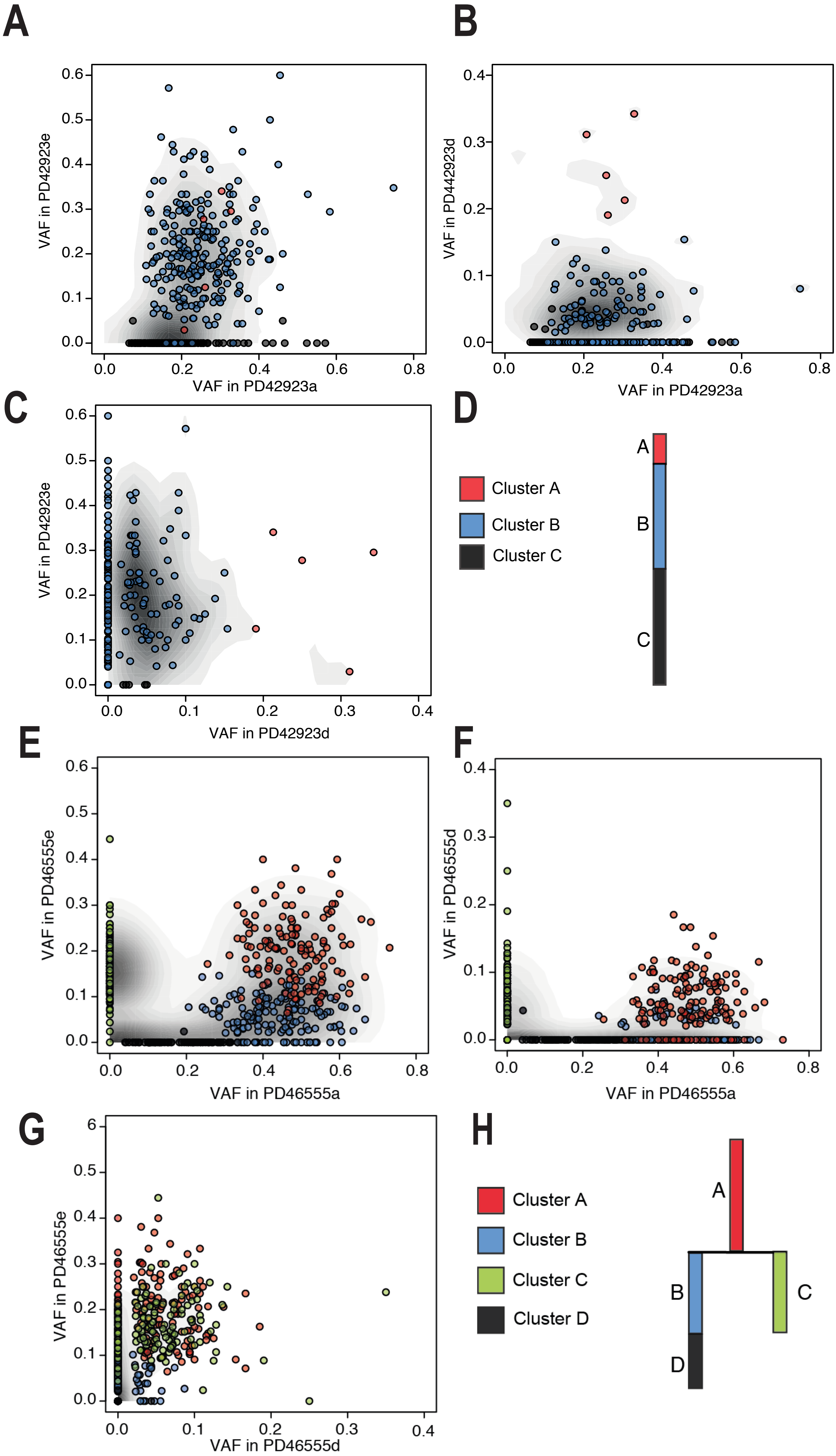
A
VAF in PD42923e
VAF in PD42923a
B
VAF in PD442923d
VAF in PD42923a
C
VAF in PD42923e
VAF in PD42923d
D
Cluster A
Cluster B
Cluster C
A
B
C
E
VAF in PD46555e
VAF in PD46555a
F
VAF in PD46555d
VAF in PD46555a
G
VAF in PD46555e
VAF in PD46555d
H
Cluster A
Cluster B
Cluster C
Cluster D
A
B
C
D

Supplementary Figure 1 | Clonal reconstruction of normal tissues in PD42923 and PD46555. **(A) - (D)** Clustering of SNVs across PD42923a, -d, and -e revealed four clusters contributing to their clonal architecture. Comparison of VAFs of these mutations between the different samples of PD42923 **(A-C)** reveals the phylogenetic ordering of these clusters **(D)**. **(E) - (H)** Clustering of SNVs across PD46555a, -d, and -e revealed four clusters contributing to their clonal architecture. Comparison of VAFs of these mutations between the different samples of PD46555 **(E-G)** reveals the phylogenetic ordering of these clusters **(H)**. The possibility that we observed tumour mutations in normal tissue because of contamination with tumour cells was addressed in three ways. Firstly, specialist pathologists reviewed sections of the pieces of tissues that we sequenced. In the hilar samples there were no tumour cells seen. In the nerve root samples, occasional tumour cells were seen. However, the frequency of these was small and could not account for the clone size observed in nerve root samples. PD46555g (cervical spine sample) was found to be heavily contaminated with tumour cells (>10%) upon inspection by the pathologists. This fraction outnumbered the INI1 negative normal cells, and hence any genomic signal of mutational sharing is likely derived from the contaminant. Therefore, we have excluded this sample from further analysis. Secondly, we performed INI1 staining to validate the expected loss of INI1 staining in normal Schwann cells, as predicted from the genomic data. This confirmed that there were morphologically normal looking Schwann cells that lacked INI1 staining in all samples that exhibited biallelic loss of *SMARCB1* genomically. Thirdly, we applied a Dirichlet process mixture modelling to define the cluster composition of the normal tissues. Tumour contamination would manifest by a single cluster, corresponding to the clonal tumour somatic mutations, being present in the normal at the contamination rate. However, we observed that the clonal tumour mutations fall into two clusters with a differential contribution to the normal tissues. This would be inconsistent with a single, clonal contaminant. In addition, such contamination would not explain mutations that are private to the normal tissues and not found in tumour tissues, of which there were many.

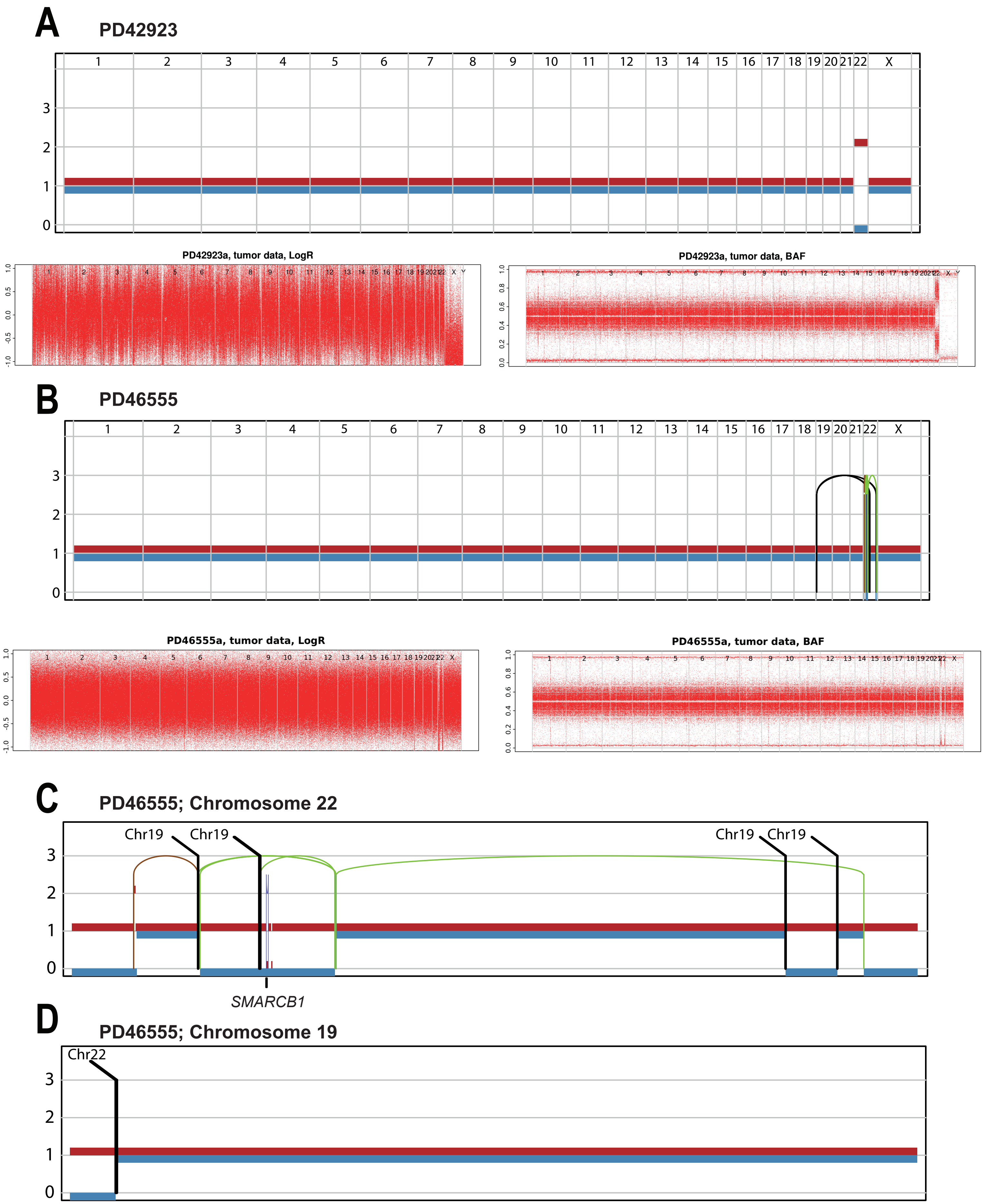
A
PD42923
1 2 3 4 5 6 7 8 9 10 11 12 13 14 15 16 17 18 19 20 21 22 X
0 1 2 3
PD42923a, tumor data, LogR
PD42923a, tumor data, BAF
B
PD46555
1 2 3 4 5 6 7 8 9 10 11 12 13 14 15 16 17 18 19 20 21 22 X
0 1 2 3
PD46555a, tumor data, LogR
PD46555a, tumor data, BAF
C
PD46555; Chromosome 22
Chr19
Chr19
Chr19
Chr19
0 1 2 3
SMARCB1
D
PD46555; Chromosome 19
Chr22
0 1 2 3

Supplementary Figure 2 | Overview of CNVs and SVs in two MRT samples.

Copy number variants (CNVs) and structural variants (SVs) for the malignant rhabdoid tumours of PD42923 **(A)** and PD46555 **(B)**, genome-wide. Major and minor copy numbers are displayed in red and blue, respectively. Black lines denote interchromosomal translocations, green inversions, brown tandem-duplications, and blue deletions. The genome-wide distributions of the log-transformed normalised read depth (logR) and B-allele frequency (BAF) are displayed below the copy number landscapes. In both cases, chromosome 22, the location of *SMARCB1*, is affected. PD42923 displays a copy number neutral loss of heterozygosity of chromosome 22. For PD46555, enhanced representations of chromosome 22 **(C)**, and chromosome 19 **(D)** reveal complex rearrangements affecting the tumour.

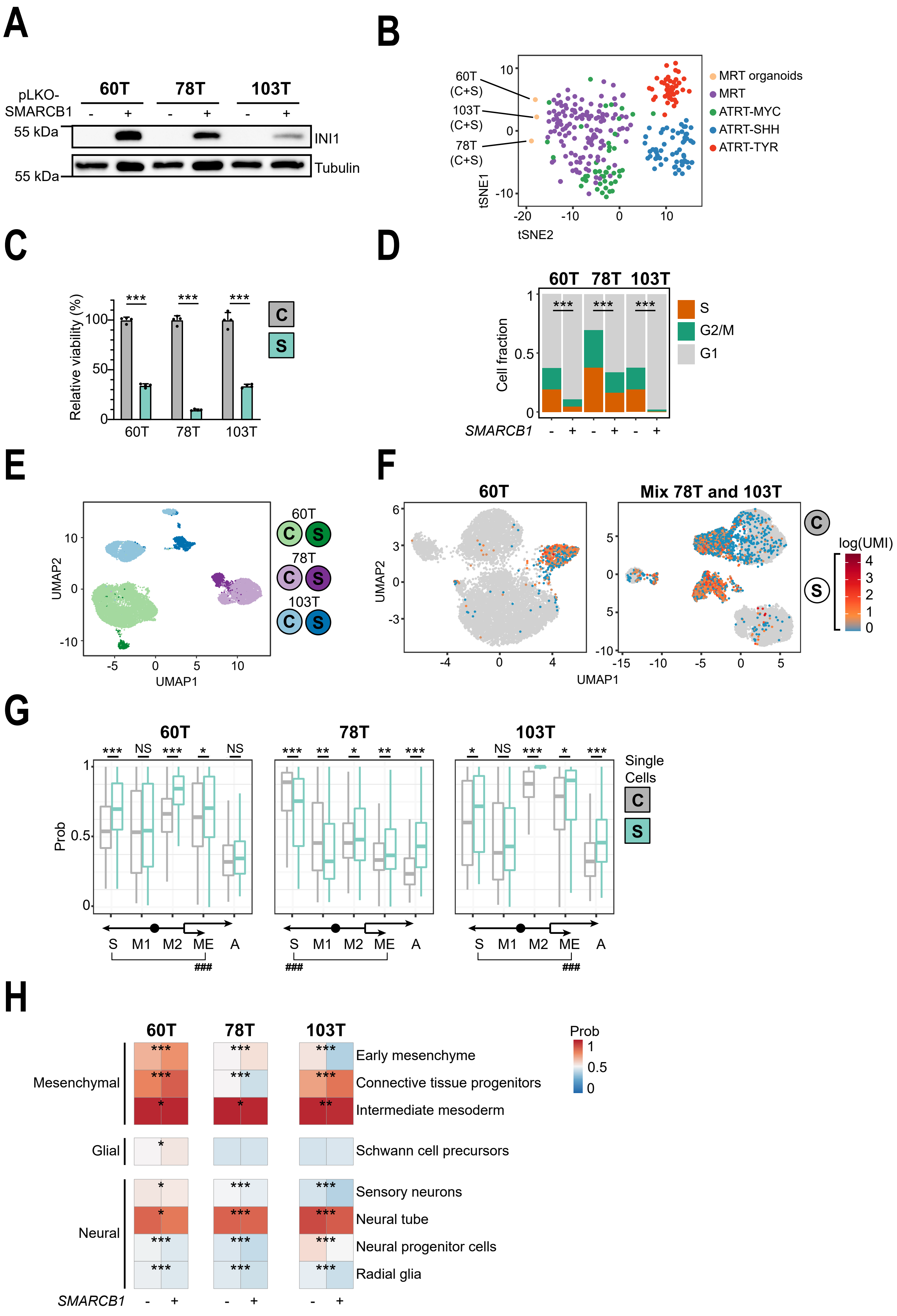
A
pLKO-
SMARCB1
60T
78T
103T
55 kDa
INI1
Tubulin
B
60T
(C+S)
103T
(C+S)
78T
(C+S)
MRT organoids
MRT
ATRT-MYC
ATRT-SHH
ATRT-TYR
tSNE1
tSNE2
C
Relative viability (%)
60T
78T
103T
***
C
S
D
60T 78T 103T
Cell fraction
S
G2/M
G1
SMARCB1
E
UMAP2
UMAP1
60T
78T
103T
C
S
F
60T
Mix 78T and 103T
UMAP2
UMAP1
log(UMI)
G
60T
78T
103T
Prob
Single
Cells
S M1 M2 ME A
###
NS
H
60T
78T
103T
Prob
Mesenchymal
Early mesenchyme
Connective tissue progenitors
Intermediate mesoderm
Glial
Schwann cell precursors
Neural
Sensory neurons
Neural tube
Neural progenitor cells
Radial glia
SMARCB1

Supplementary Figure 3 | *SMARCB1* reconstitution in organoids as model for MRT differentiation.

(**A)** Western blot analysis of INI1 expression in MRT organoids lentivirally transduced with either a control (-) or *SMARCB1* expression (+) plasmid. Tubulin protein levels are used as loading control. Molecular weight markers are indicated in kilodalton (kDa). Source data are provided as a Source Data file. **(B)** t-SNE representation of DNA methylation profiles of MRT control (C) and *SMARCB1*+ (S) organoids compared to MRT and AT/RT (subtypes MYC, SHH, TYR) tissue, showing that MRT organoids cluster closely to MRT tissue independent of *SMARCB1* expression. t-SNE coordinates of MRT control and *SMARCB1*+ organoids completely overlap. Different patient lines are indicated. **(C)** Bar graphs represent cell viability of MRT *SMARCB1*+ (green) relative to MRT control organoids (gray). Mean and SD (error bars) of 4 technical replicates (dot) are indicated. Source data are provided as a Source Data file. Growth was assessed by comparing control to *SMARCB1*+ organoids. P-values were calculated using an unpaired Student's t test (two-tailed): * <0.05, ** <0.01, *** <0.001 (p-value: 60T=2.1e-8, 78T=1.4e-8, 103T=2.5e-6). **(D)** Stacked bar plot represents relative frequencies of single cells from MRT control (-) or *SMARCB1*+ (+) organoids annotated for cell cycle phase (derived from single cell transcriptomes). Colours distinguish G1 (gray), G2/M (green) or S (orange) phase. Frequencies of cell cycle phase annotations were compared between control and *SMARCB1*+ organoids for each patient line. P-values were calculated using a chi-square test (two-tailed): *** <1e-15 (-log10(p-value): 60T=27, 78T=77, 103T=96). **(E)** UMAP representation of single cells from MRT organoid lines 60T (green, control/*SMARCB1*+: 8059/425 cells), 78T (purple, control/*SMARCB1*+: 3195/806 cells) and 103T (blue, control/*SMARCB1*+: 2694/953 cells) distinguishing control (light) and *SMARCB1*+ (dark) cells. **(F)** UMAP representation of single cells separated by sample: 60T (control/*SMARCB1*+: 8059/638 cells) or mix 78T and 103T (control/*SMARCB1*+: 7214/3389 cells; demultiplexing was performed as described in Methods). For these UMAPs, cells were not filtered for *SMARCB1* expression and thus include non-transduced cells, demonstrating that unsuccessfully transduced cells in the *SMARCB1*+ sample cluster with control-transduced cells thereby excluding batch effects. Colour distinguishes the batch of MRT control cells (gray) from the batch of MRT *SMARCB1*+ cells (colour-code from blue to red refers to *SMARCB1* transcript levels (unique molecular identifier (UMI)). *SMARCB1*-negative cells were filtered out from *SMARCB1*+ samples for all subsequent analysis. **(G)** Box plots represent single cell similarity

scores (n = 60T control/*SMARCB1*+: 8059/425 cells; 78T control/*SMARCB1*+: 3195/806 cells; 103T control/*SMARCB1*+: 2694/953 cells) for cell types of the mesenchyme/autonomic (ME/A) or sensory (S) branch (illustrated in Fig. 2a). Box plots indicate median (middle line), 25th and 75th percentile (box). Whiskers represent the range excluding outliers. Mesenchyme and sensory similarity were compared for control cells to identify the major neural crest cell type signal at baseline. P-values were calculated using a paired Student's t test (two-tailed): ### <1e-15 below figure to indicate cell type with highest average similarity score (-log10(p-value): 60T=100, 78T=Inf, 103T=52). Additionally, similarity scores were compared between control and *SMARCB1*+ cells. P-values were calculated using an unpaired Student's t test (two-tailed): * <1e-3, ** <1e-9, *** <1e-15 (exact p-values are indicated in Supplementary Table 4). **(H)** Heatmaps represent similarity of MRT control (-) and *SMARCB1*+ (+) cells to a mouse organogenesis cell type reference[19], comparing early mesenchymal and neural cell type similarities. Colours represent the average probability (prob) that the MRT cells are similar to the indicated cell type (predicted similarity score estimated by logistic regression[12]). Changes in similarity scores between control and *SMARCB1*+ cells were assessed. P-values were calculated using an unpaired Student's t test (two-tailed): * <1e-3, ** <1e-9, *** <1e-15 (exact p-values are indicated in Supplementary Table 4).

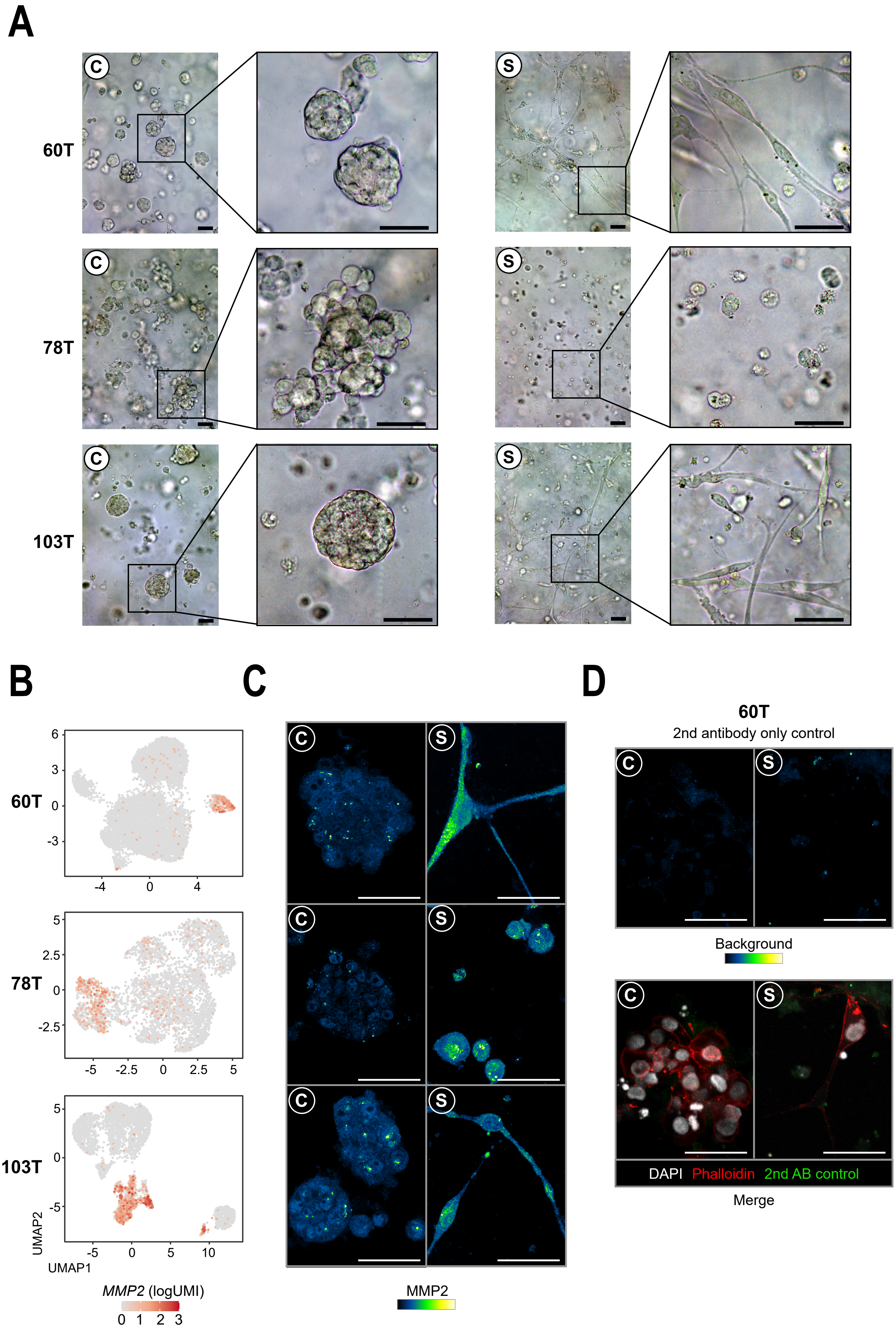
A
60T
78T
103T
C
S
B
60T
78T
103T
UMAP1
UMAP2
MMP2 (logUMI)
0 1 2 3
C
MMP2
D
60T
2nd antibody only control
Background
DAPI Phalloidin 2nd AB control
Merge

Supplementary Figure 4 | Morphological transformation of MRT organoids upon *SMARCB1* reconstitution.

(A) Representative brightfield images of MRT control (C) and *SMARCB1*+ (S) organoid lines including zoom in. Scale bars equal 100 µm. **(B)** UMAP representation of single cells from MRT control and *SMARCB1*+ organoid lines. Colour-code from gray to red refers to *MMP2* transcript levels (unique molecular identifier (UMI)). **(C)** Representative immunofluorescence images of MRT control (C) and *SMARCB1*+ (S) organoids stained for MMP2. Merged images are shown in Fig. 2b. Colour-code represents immunofluorescent signal intensity. Scale bars equal 50 µm. **(D)** Representative immunofluorescence images of 60T control (C) and *SMARCB1*+ (S) organoids only incubated with the secondary antibody, to determine background signal (top panel). Colour-code represents immunofluorescent signal intensity. Bottom panel shows a merge of DAPI (white; nuclei), phalloidin (red; membranes) and background signal (green). Scale bars equal 50 µm.

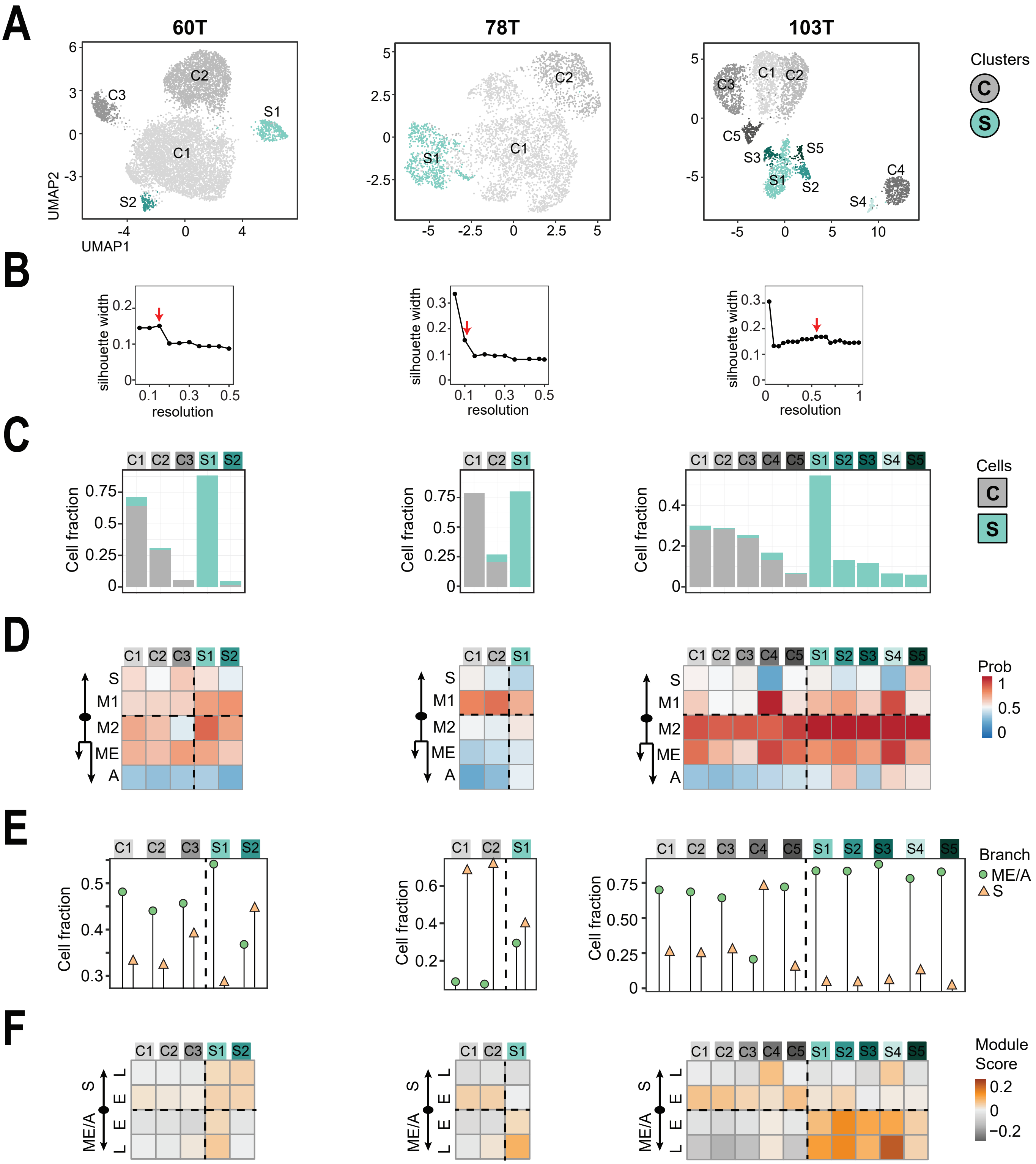
A
60T
78T
103T
Clusters
C
S
UMAP2
UMAP1
C1
C2
C3
C4
C5
S1
S2
S3
S4
S5
B
silhouette width
resolution
C
Cell fraction
Cells
D
Prob
S
M1
M2
ME
A
E
Branch
ME/A
S
F
Module Score
L
E

Supplementary Figure 5 | Intra-organoid heterogeneity of neural crest signals.

(A) UMAP representation of single cells from MRT control and *SMARCB1+* organoid lines showing cluster assignment. Cells are coloured for either control (gray: light to dark) or *SMARCB1+* (green: light to dark) dominant clusters, which are numbered according to cluster size. (60T C1-3/S1-2: 5,209/2,350/425/375/125 cells, 78T C1-2/S1: 2,624/725/652 cells, 103T C1-5/S1-5: 769/768/664/395/172/521/126/110/64/58 cells) **(B)** Scatter plot depicts a series of resolutions used for the Louvain-based clustering of MRT control and *SMARCB1+* single cells, and the corresponding quantification of average silhouette width, which was used to determine optimal clustering resolution. The resolution with the highest average silhouette width (arrow) was used for subsequent analyses. **(C)** Stacked bar plots represent relative frequencies of control (gray) and *SMARCB1+* (green) single cells for each cluster, showing a segregation of cells upon *SMARCB1* reconstitution. **(D)** Heatmaps show average similarity score per cluster dominant for either MRT control (gray) or *SMARCB1+* (green) cells. Clusters were compared to cell types from the mesenchymal/autonomic or sensory branch, showing heterogeneity of neural crest signals within and between patient lines. Abbreviations are indicated in Fig. 2a. **(E)** Graphs represent relative frequencies of cell type annotations for MRT control (gray) or *SMARCB1+* (green) dominant clusters. Cell type annotation was assigned for each single cell based on the highest similarity score. Cell type annotations were grouped into the mesenchymal/autonomic (ME/A; green circle) or sensory (S; orange triangle) differentiation branch. **(F)** Heatmaps represent average gene module scores for MRT control (gray) or *SMARCB1+* (green) dominant clusters. Module scores were generated by averaging gene expression levels per set of genes. Gene sets include marker genes for either sensory (S) or mesenchymal/autonomic (ME/A) differentiation branches, distinguishing early (E) and late (L) differentiation genes.

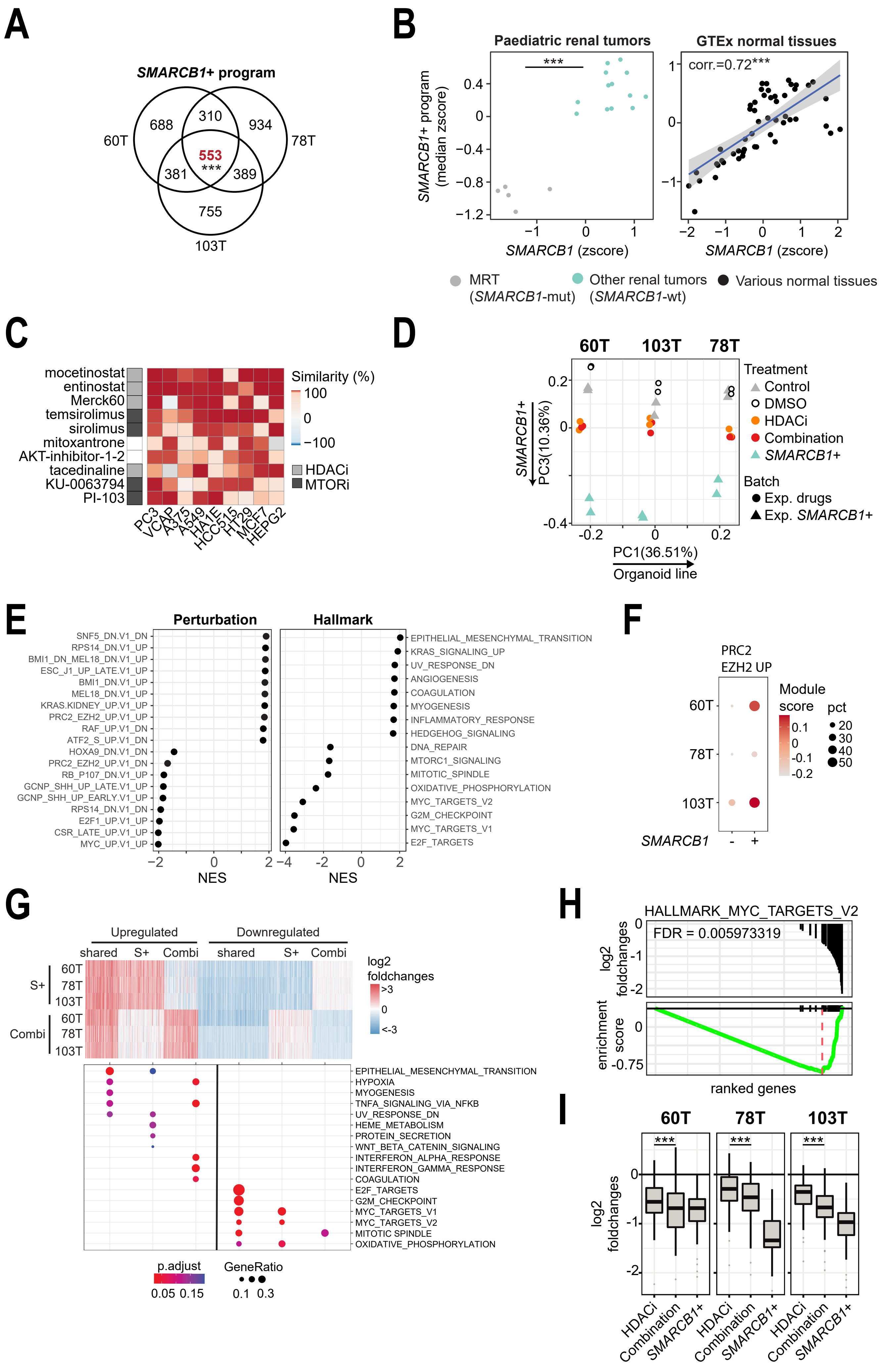

A
SMARCB1+ program
60T
78T
103T
688
310
934
553
***
381
389
755
B
Paediatric renal tumors
GTEx normal tissues
SMARCB1+ program (median zscore)
***
corr.=0.72***
SMARCB1 (zscore)
SMARCB1 (zscore)
MRT (SMARCB1-mut)
Other renal tumors (SMARCB1-wt)
Various normal tissues
C
mocetinostat
entinostat
Merck60
temsirolimus
sirolimus
mitoxantrone
AKT-inhibitor-1-2
tacedinaline
KU-0063794
PI-103
PC3
VCAP
A375
A549
HA1E
HCC515
HT29
MCF7
HEPG2
Similarity (%)
100
0
−100
HDACi
MTORi
D
60T
103T
78T
SMARCB1+ PC3(10.36%)
PC1(36.51%)
Organoid line
Treatment
Control
DMSO
HDACi
Combination
SMARCB1+
Batch
Exp. drugs
Exp. SMARCB1+
E
Perturbation
Hallmark
SNF5_DN.V1_DN
RPS14_DN.V1_UP
BMI1_DN_MEL18_DN.V1_UP
ESC_J1_UP_LATE.V1_UP
BMI1_DN.V1_UP
MEL18_DN.V1_UP
KRAS.KIDNEY_UP.V1_UP
PRC2_EZH2_UP.V1_UP
RAF_UP.V1_DN
ATF2_S_UP.V1_DN
HOXA9_DN.V1_DN
PRC2_EZH2_UP.V1_DN
RB_P107_DN.V1_UP
GCNP_SHH_UP_LATE.V1_UP
GCNP_SHH_UP_EARLY.V1_UP
RPS14_DN.V1_DN
E2F1_UP.V1_UP
CSR_LATE_UP.V1_UP
MYC_UP.V1_UP
EPITHELIAL_MESENCHYMAL_TRANSITION
KRAS_SIGNALING_UP
UV_RESPONSE_DN
ANGIOGENESIS
COAGULATION
MYOGENESIS
INFLAMMATORY_RESPONSE
HEDGEHOG_SIGNALING
DNA_REPAIR
MTORC1_SIGNALING
MITOTIC_SPINDLE
OXIDATIVE_PHOSPHORYLATION
MYC_TARGETS_V2
G2M_CHECKPOINT
MYC_TARGETS_V1
E2F_TARGETS
NES
NES
F
PRC2 EZH2 UP
60T
78T
103T
Module score
pct
SMARCB1
G
Upregulated
Downregulated
shared
S+
Combi
S+
Combi
log2 foldchanges
>3
0
<-3
EPITHELIAL_MESENCHYMAL_TRANSITION
HYPOXIA
MYOGENESIS
TNFA_SIGNALING_VIA_NFKB
UV_RESPONSE_DN
HEME_METABOLISM
PROTEIN_SECRETION
WNT_BETA_CATENIN_SIGNALING
INTERFERON_ALPHA_RESPONSE
INTERFERON_GAMMA_RESPONSE
COAGULATION
E2F_TARGETS
G2M_CHECKPOINT
MYC_TARGETS_V1
MYC_TARGETS_V2
MITOTIC SPINDLE
OXIDATIVE_PHOSPHORYLATION
p.adjust
0.05 0.15
GeneRatio
0.1 0.3
H
HALLMARK_MYC_TARGETS_V2
FDR = 0.005973319
log2 foldchanges
enrichment score
ranked genes
I
60T
78T
103T
***
log2 foldchanges
HDACi
Combination
SMARCB1+

Supplementary Figure 6 | Finding drugs mimicking *SMARCB1*-induced MRT differentiation.

(A) Venn diagram showing the overlap of genes (red) upregulated by *SMARCB1* in MRT organoids, further referred to as the *SMARCB1*+ program. The intersect was assessed for three independent patient lines. P-value was calculated using a multi-set exact test (one-tailed)[48]: *** <1e-15 (p-value: 0) **(B)** Scatter plot showing the relative expression (median z-score) of the *SMARCB1*+ program versus *SMARCB1* expression levels (z-score) for tissues from a paediatric renal tumour biobank[17] (left) or normal tissues (right). The expression of the *SMARCB1*+ program was compared between *SMARCB1* mutant (mut) tissues (MRT; gray; n = 5) and *SMARCB1* wild-type tissues (wt; other renal tumours; green; n = 13). P-values were calculated using an unpaired Student's t test (two-tailed): *** <0.001 (p-value=5.5e-10). In normal tissues (black), the relationship of *SMARCB1* expression levels and the *SMARCB1*+ program was assessed. Correlation coefficient (corr.) and p-value were calculated using Pearson's correlation: *** <0.001 (p-value=2.2e-9). **(C)** Represented is the top 10 of drugs that mimic mRNA changes of *SMARCB1* re-expression, extracted from the CLUE database[20]. Colour-code represents the percentage of similarity for each cell line (x-axis) and drug treatment (y-axis) to *SMARCB1* re-expression. Drugs are annotated as HDAC (dark-gray) or mTOR (light-gray) inhibitors. **(D)** Principal component analysis (PCA) of bulk mRNA-seq samples shows the transcriptional effect of indicated drug treatment (different colours) on MRT organoids, compared with MRT control (gray) or *SMARCB1*+ (green) organoids (n = 2 independent experiments). PC1 and PC3 separate samples by organoid line and drug treatment/*SMARCB1* re-expression. Batch refers to either the experiment with *SMARCB1* re-expression (*SMARCB1*+) or drug treatment (drugs). **(E)** Gene set enrichment analysis (GSEA) for hallmark and perturbation gene sets, using genes ranked by the mRNA changes induced by *SMARCB1*. The top 10 of statistically significant gene sets (adjusted p-value <0.01) is presented for up- and downregulated genes, showing the normalized enrichment score (NES) on the x-axis. P-values were calculated using a permutation test (two-tailed) and were corrected for multiple-testing. **(F)** Genes repressed by polycomb repressive complex subunit EZH2 were upregulated by *SMARCB1* (Supplementary Fig. 6e). This result was confirmed in the scRNA-seq experiment. Dot plot shows gene module scores (average gene expression level for PRC2_EZH2 gene set) for MRT control (-) and *SMARCB1*+ (+) organoids for each patient line. Colour-code from gray to red refers to average module score. Dot size refers to the percentage of

cells (pct) expressing the gene module. **(G)** Enrichment analysis for hallmark pathways using unordered sets of differentially expressed genes upon *SMARCB1* re-expression (S+) or combination treatment, either shared or exclusive. The top 5 of significantly enriched terms (adjusted p-value <0.25) are presented (bottom). P-values were calculated using a Fischer's exact test (one-tailed) and were corrected for multiple-testing. Changes in mRNA levels for these genes are visualized in the heatmap (top) for each patient line. **(H)** Gene set enrichment analysis for MYC target genes, with bars representing average mRNA changes of *SMARCB1* re-expression in MRT organoids (top). Enrichment score is visualized by the ranking of genes based on these mRNA changes, showing enrichment for MYC targets with genes downregulated by *SMARCB1* re-expression (bottom). **(I)** Boxplots represent mRNA expression changes of MYC target genes (n = 58 genes) induced by either drug treatment or *SMARCB1* re-expression for each MRT organoid line. Box plots indicate median (middle line), 25th and 75th percentile (box). Whiskers represent the range excluding outliers (dot). Additional effect of combination treatment on MYC target genes (n=58 genes) was assessed by comparing combination with HDACi treatment. P-values were calculated using a paired Student's t test (two-tailed): *** <0.001 (p-value: 60T=4.9e-6, 78T=9.7e-10, 103T=2.6e-14).

A

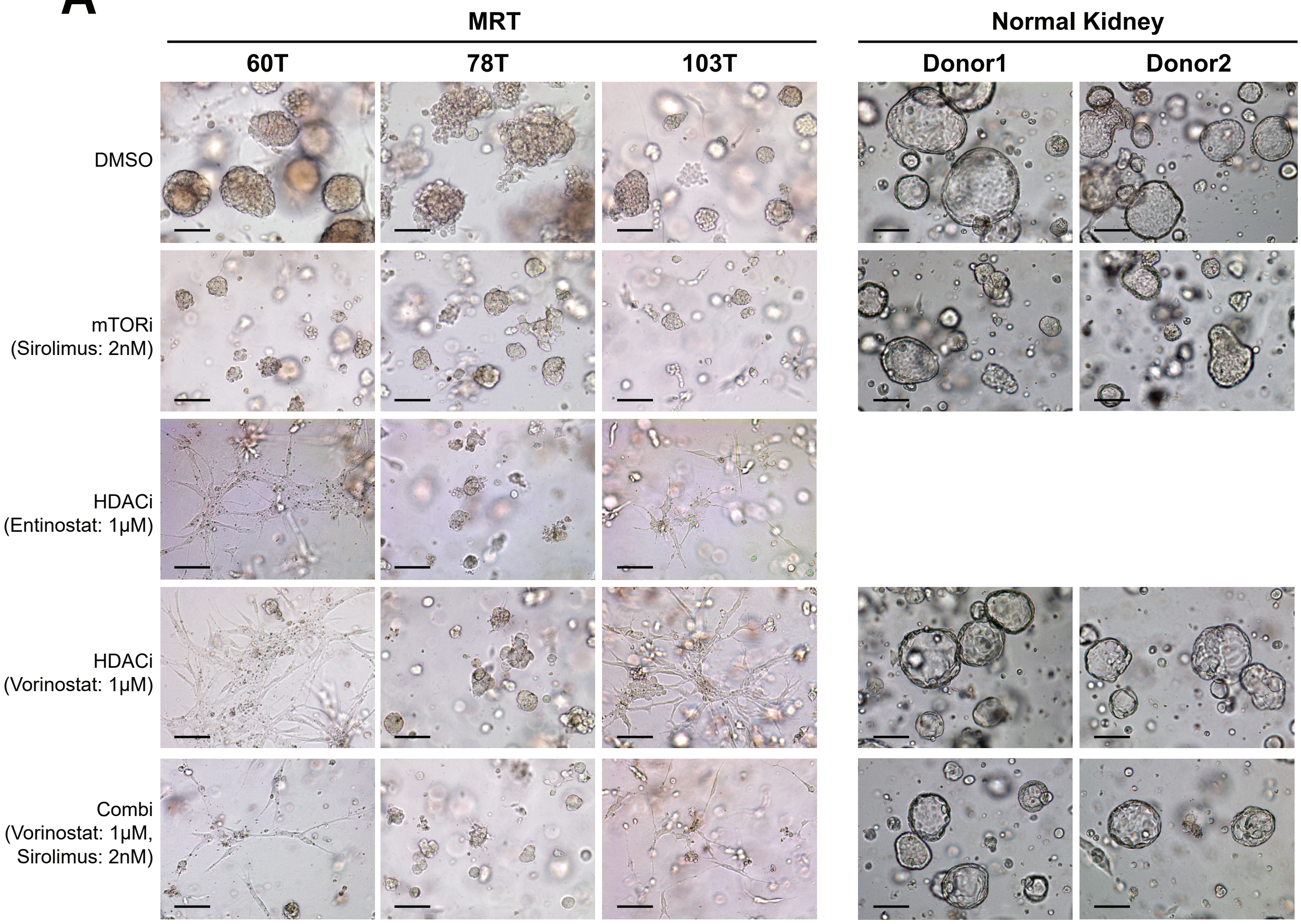
MRT
Normal Kidney
60T
78T
103T
Donor1
Donor2
DMSO
mTORi
(Sirolimus: 2nM)
HDACi
(Entinostat: 1μM)
HDACi
(Vorinostat: 1μM)
Combi
(Vorinostat: 1μM,
Sirolimus: 2nM)

B

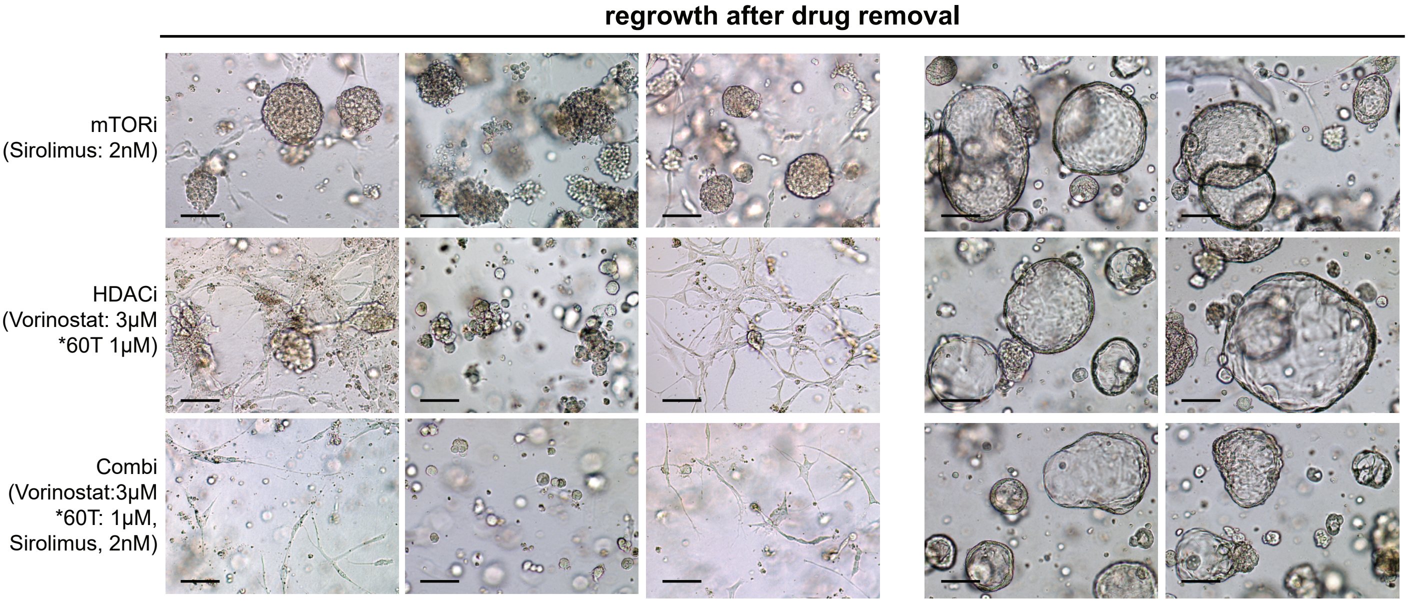
regrowth after drug removal
mTORi
(Sirolimus: 2nM)
HDACi
(Vorinostat: 3μM
*60T 1μM)
Combi
(Vorinostat:3μM
*60T: 1μM,
Sirolimus, 2nM)

Supplementary Figure 7 | HDACi and MTORi combination treatment recapitulates MRT *SMARCB1+* organoid morphology.

(A) Representative brightfield images of MRT and normal kidney organoid lines treated with vehicle (DMSO), sirolimus (2nM; mTORi), entinostat (1µM; HDACi), vorinostat (1µM; HDACi) or the combination of sirolimus (2nM) and vorinostat (1µM). Scale bars denote 100 µm. **(B)** Representative brightfield images of MRT and normal kidney organoid lines after wash-out of drugs (T2) showing that combination treatment induces a durable differentiation phenotype in MRT. Scale bars denote 100 µm.

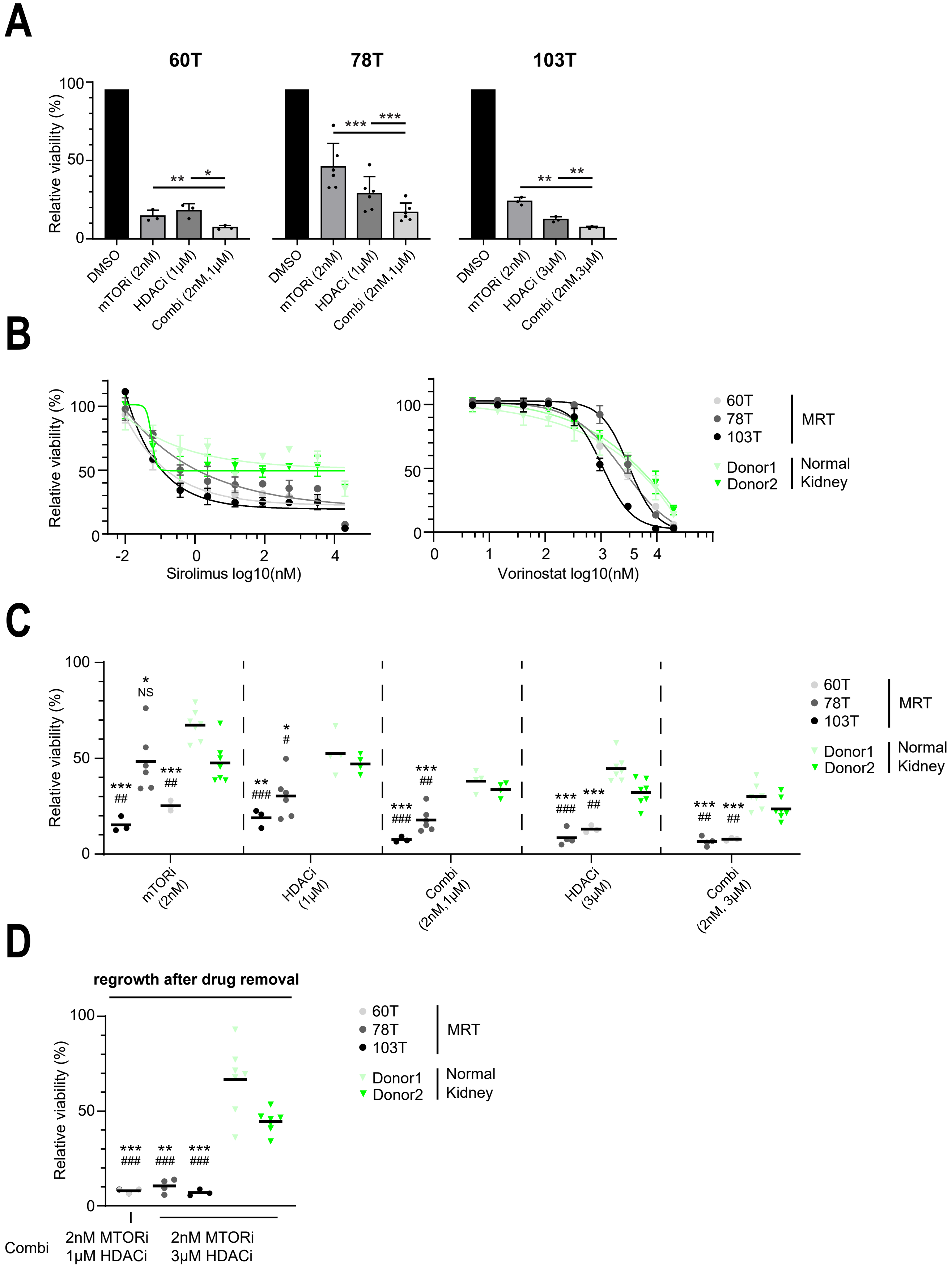
A
60T
78T
103T
Relative viability (%)
DMSO
mTORi (2nM)
HDACi (1µM)
Combi (2nM,1µM)
HDACi (3µM)
Combi (2nM,3µM)
B
Relative viability (%)
Sirolimus log10(nM)
Vorinostat log10(nM)
60T
78T
103T
MRT
Donor1
Donor2
Normal
Kidney
C
Relative viability (%)
mTORi (2nM)
HDACi (1µM)
Combi (2nM,1µM)
HDACi (3µM)
Combi (2nM, 3µM)
D
regrowth after drug removal
Relative viability (%)
Combi
2nM MTORi
1µM HDACi
2nM MTORi
3µM HDACi

Supplementary Figure 8 | Growth inhibitory effects of HDACi and MTORi are MRT specific
(A) Bar graphs represent the cell viability of MRT organoids after drug treatment (T1) relative to vehicle control (DMSO). Mean and SD (error bars) of independent experiments (dot) are indicated (n = 60T/103T: 3; 78T: 6). Each independent experiment is an average of 4 technical replicates. Source data are provided as a Source Data file. The additional effect of drug combination is assessed by comparing combination treatment with either mTORi (2nM) or HDACi (1µM or 3µM). P-values were calculated using an unpaired Student's t test (two-tailed): * <0.05, ** < 0.01, *** <0.001. (p-value: Combi vs mTORi 60T=0.008, 78T=4.5e-7, 103T=0.003; vs HDACi 60T=0.02, 78T=0.0002, 103T=0.005) **(B)** Dose response curves of sirolimus (mTORi, left) and vorinostat (HDACi, right) on MRT (gray) and normal kidney (green) organoid lines. Each dot and error bar represent the mean and SD of two independent experiments (each independent experiment is an average of 4 technical replicates). Source data are provided as a Source Data file. **(C)** Graph shows the cell viability of MRT (gray) and normal kidney (green) organoids after drug treatment (T1) relative to vehicle control (DMSO). Organoids were treated with either sirolimus (2nM), vorinostat (1µM or 3µM) or the combination. Each dot represents an independent experiment (n = 60T/103T: 3, 78T mTOR/HDAC1µM/Combi2nM1µM: 6, 78T HDAC3µM/Combi2nM3µM: 4, normal kidney: 7), which is an average of 4 technical replicates. Source data are provided as a Source Data file. The mean is indicated as a horizontal line for each organoid line. Average cell viability of MRT and normal kidney organoids were compared. P-values were calculated using an unpaired Student's t test (two-tailed): test with donor 1 * <0.05, ** < 0.01, *** <0.001, test with donor 2 # <0.05, ## < 0.01, ### <0.001 (exact p-values are indicated in Supplementary Table 4). **(D)** Graph shows the cell viability of MRT and normal kidney organoids after drug washout (T2) normalized to timepoint 1 (T1) DMSO controls. Each dot represents an independent experiment (n = 60T/103T: 3, 78T: 4, normal kidney: 7), which is an average of 4 technical replicates. Source data are provided as a Source Data file. The mean is indicated as a horizontal line for each organoid line. Regrowth capability was assessed by comparison of average cell viability of MRT and normal kidney organoids. P-values were calculated using an unpaired Student's t test (two-tailed): test with donor 1 * <0.05, ** < 0.01, *** <0.001, test with donor 2 # <0.05, ## < 0.01, ### <0.001 (p-value: Donor1 vs 60T=0.0007, vs 78T=0.0002, vs 103T=0.0006; Donor2 vs 60T=2.9e-6, vs 78T=1.3e-6, vs 103T=2.4e-6).

Case	Sample	Sample type	FFPE	WGS Coverage	Platform
PD42923	PD42923a	Tumour	N	43.68	X10
PD42923	PD42923b	Blood	N	31.46	X10
PD42923	PD42923c	Kidney	N	31.74	X10
PD42923	PD42923d	Ganglion	Y	25.47	X10
PD42923	PD42923e	Nerve	Y	26.19	X10
PD42923	PD42923f	Tumour	Y	22.22	X10
PD42923	PD42923g	Kidney	Y	25.61	X10
PD46555	PD46555a	Tumour	N	36.98	Novaseq
PD46555	PD46555d	Dorsal nerve roots	N	31.34	Novaseq
PD46555	PD46555e	Ventral nerve roots	N	36.65	Novaseq
PD46555	PD46555f	Spinal cord	N	35.86	Novaseq
PD46555	PD46555g	Upper cervical spine	N	27.26	Novaseq
PD46555	PD46555h	Spinal cord	N	29.7	Novaseq
PD46555	PD46555i	Posterior dura	N	40.78	Novaseq
PD46555	PD46555j	Fat	N	28.48	Novaseq
PD46555	PD46555k	Fat	N	34.98	Novaseq
PD46555	PD46555l	Muscle	N	34.45	X10
PD46555	PD46555m	Muscle	N	29.79	Novaseq
PD46555	PD46555r	Skin	N	39.51	Novaseq
PD46555	PD46555s	Skin	N	30.62	Novaseq
PD46555	PD46555w	Blood	N	30.77	Novaseq

Supplementary Table 1 | Overview of WGS data.

Patient line	Diagnosis	Source
60T	Malignant Rhabdoid Tumor of the Kidney	Primary Tumor
78T	Malignant Rhabdoid Tumor of the Kidney	Primary Tumor
103T	Malignant Rhabdoid Tumor of the Kidney	Primary Tumor

Supplementary Table 2 | MRT organoid features.

Sample name	Original ID	Patient line	Details	Gender	10X kit	average reads/cell
MRT_60T_CTRL	5640STDY7891142	60T	MRT Control	male	3' v2	35970
MRT_60T_SMB	5640STDY7891143	60T	MRT *SMARCB1* +	male	3' v2	490249
MRT_78and103_CTRL	snRNA10x-Lars-CTRL-17579	78T and 103T mixed	MRT Control	78T=male,103T=female	3' v3	34798
MRT_78and103_SMB	snRNA10x-Lars-SMB1-17580	78T and 103T mixed	MRT *SMARCB1* +	78T=male,103T=female	3' v3	44561

Sample name	sequencing platform	Genome reference	cellranger version	%mito	SMARCB1 filter	gene cutoff
MRT_60T_CTRL	Illumina HiSeq 4000	GRCh38 1.2.0	2.0.2	10	SMARCB1 > 0	none
MRT_60T_SMB	Illumina HiSeq 4000	GRCh38 1.2.0	2.0.2	10	SMARCB1 == 0	none
MRT_78and103_CTRL	NextSeq 500	GRCh38 1.2.0	3.0.3	20	SMARCB1 > 0	1500
MRT_78and103_SMB	NextSeq 500	GRCh38 1.2.0	3.0.3	20	SMARCB1 == 0	1500

Sample name	EGA ID
MRT_60T_CTRL	EGAN00002133134
MRT_60T_SMB	EGAN00002133135
MRT_78and103_CTRL	EGAN00002580391, EGAN00002580392, EGAN00002580393, EGAN00002580394
MRT_78and103_SMB	EGAN00002580397, EGAN00002580398

Supplementary Table 3 | Sample processing info scRNA-seq.

Supplementary Fig. 3g	SMARCB1 vs Control		
Cell Type	60T	78T	103T
Sensory	4.58133E-28	3.14728E-22	1.75E-08
Migratory.1	0.2275684	3.315E-15	0.08191271
Migratory.2	5.89544E-67	0.00003463	0
Mesenchyme	0.0002235	4.20523E-12	0.0006274
Autonomic	0.02985853	7.49681E-93	1.3824E-57
Supplementary Fig. 3h	SMARCB1 vs Control		
Cell Type	60T	78T	103T
Early mesenchyme	1.48789E-35	2.00176E-18	4.454E-173
Connective tissue progenitors	1.59887E-80	2.41191E-68	2.6248E-127
Intermediate mesoderm	0.01167182	6.39985E-07	3.7136E-12
Schwann cell precursors	5.15326E-07	0.3710319	0.4429936
Sensory neurons	0.000473307	1.88498E-23	6.4646E-141
Neural tube	0.007759082	1.6638E-27	7.40169E-21
Neural progenitor cells	7.6889E-15	1.73252E-35	5.59331E-73
Radial glia	1.20211E-16	1.41149E-24	6.9577E-111
Supplementary Fig. 8c	Comparison vs normal kidney donor 1		
Drug	60T	78T	103T
mTORi (2nM)	5.78324E-06	0.016856109	2.35666E-05
HDACi (1μM)	0.003957308	0.014547196	NA
Combi (2nM, 1μM)	0.000197062	0.000667108	NA
HDACi (3μM)	NA	5.37192E-06	5.74512E-05
Combi (2nM, 3μM)	NA	9.53401E-05	0.000535419
	Comparison vs normal kidney donor 2		
Drug	60T	78T	103T
mTORi (2nM)	0.001134997	0.927179112	0.008510203
HDACi (1μM)	0.000573236	0.025865327	NA
Combi (2nM, 1μM)	9.5596E-05	0.001887569	NA
HDACi (3μM)	NA	0.000168	0.001769478
Combi (2nM, 3μM)	NA	0.000388825	0.001892323

Supplementary Table 4 | Exact p-values.
