# Supplementary material for: Somatic mutations and single-cell transcriptomes reveal the root of malignant rhabdoid tumours
Source: Nat Commun. 2021 Mar 3;12:1407. doi: 10.1038/s41467-021-21675-6 (PMC7930245; doi:10.1038/s41467-021-21675-6)
Supplement: Supplementary file 2 — Description of Additional Supplementary Files [file 41467_2021_21675_MOESM2_ESM.docx]

**Description of Additional Supplementary Files**

File Name: Supplementary Data 1

Description: Single nucleotide variants (SNVs), copy number variants (CNVs) and structural variants called in PD42923 and PD46555.

File Name: Supplementary Data 2

Description: mRNA changes upon SMARCB1 reconstitution in MRT single cell clusters.

File Name: Supplementary Data 3

Description: Genes differentially expressed upon SMARCB1 re-expression and drug treatment in MRT organoids.
