# Supplementary material for: Somatic mutations and single-cell transcriptomes reveal the root of malignant rhabdoid tumours
Source: Nat Commun. 2021 Mar 3;12:1407. doi: 10.1038/s41467-021-21675-6 (PMC7930245; doi:10.1038/s41467-021-21675-6)
Supplement: Supplementary file 6 — Reporting Summary [file 41467_2021_21675_MOESM6_ESM.pdf]

nature research

Corresponding author(s): Sam Behjati and Jarno Drost

Last updated by author(s): Jan 22, 2021

Reporting Summary

Nature Research wishes to improve the reproducibility of the work that we publish. This form provides structure for consistency and transparency in reporting. For further information on Nature Research policies, see our Editorial Policies and the Editorial Policy Checklist.

Statistics

For all statistical analyses, confirm that the following items are present in the figure legend, table legend, main text, or Methods section.

n/a	Confirmed	
☐	☒	The exact sample size (*n*) for each experimental group/condition, given as a discrete number and unit of measurement
☐	☒	A statement on whether measurements were taken from distinct samples or whether the same sample was measured repeatedly
☐	☒	The statistical test(s) used AND whether they are one- or two-sided<br>*Only common tests should be described solely by name; describe more complex techniques in the Methods section.*
☒	☐	A description of all covariates tested
☐	☒	A description of any assumptions or corrections, such as tests of normality and adjustment for multiple comparisons
☐	☒	A full description of the statistical parameters including central tendency (e.g. means) or other basic estimates (e.g. regression coefficient) AND variation (e.g. standard deviation) or associated estimates of uncertainty (e.g. confidence intervals)
☐	☒	For null hypothesis testing, the test statistic (e.g. *F*, *t*, *r*) with confidence intervals, effect sizes, degrees of freedom and *P* value noted<br>*Give P values as exact values whenever suitable.*
☒	☐	For Bayesian analysis, information on the choice of priors and Markov chain Monte Carlo settings
☐	☒	For hierarchical and complex designs, identification of the appropriate level for tests and full reporting of outcomes
☐	☒	Estimates of effect sizes (e.g. Cohen's *d*, Pearson's *r*), indicating how they were calculated

Our web collection on statistics for biologists contains articles on many of the points above.

Software and code

Policy information about availability of computer code

Data collection	See below.
Data analysis	BWA (0.7.17; https://github.com/lh3/bwa)<br>CaVEMan (1.14.0; https://github.com/cancerit)<br>ASCAT (4.3.3; https://github.com/cancerit)<br>Battenberg (3.5.2; https://github.com/cancerit)<br>BRASS (6.3.0; https://github.com/cancerit)<br>Cellranger (2.0.2; https://github.com/10XGenomics/cellranger)<br>Cellranger (3.0.3; https://github.com/10XGenomics/cellranger)<br>scanpy (1.4.4.post1; https://github.com/theislab/scanpy)<br>demuxlet (downloaded from Github in Oct.2020; https://github.com/statgen/demuxlet)<br>scrublet (0.2.1; https://github.com/AllonKleinLab/scrublet)<br>biomaRt (2.40.4; https://github.com/grimbough/biomaRt)<br>glmnet (4.0.2; https://github.com/cran/glmnet)<br>Seurat (3.1.4; https://github.com/satijalab/seurat)<br>sctransform (0.2.1; https://github.com/ChristophH/sctransform)<br>Trimmomatic (0.36.5; https://github.com/timflutre/trimmomatic)<br>STAR (2.6.0; https://github.com/alexdobin/STAR)<br>featureCounts (1.6.3; https://github.com/byee4/featureCounts)<br>DESeq2 (1.22.1; https://github.com/mikelove/DESeq2)<br>limma (3.38.3; https://github.com/cran/limma)<br>pheatmap (1.0.12; https://github.com/raivokolde/pheatmap)

clusterProfiler (3.10.1; https://github.com/YuLab-SMU/clusterProfiler)
GraphPad Prism (8.0.2)
synergyfinder (1.8.0; https://github.com/hly89/synergyfinder)

Further details on the pipelines used for data analysis can be found in the Methods section and on the following page: https://github.com/kheleon/mrt-paper

For manuscripts utilizing custom algorithms or software that are central to the research but not yet described in published literature, software must be made available to editors and reviewers. We strongly encourage code deposition in a community repository (e.g. GitHub). See the Nature Research guidelines for submitting code & software for further information.

Data

Policy information about availability of data

All manuscripts must include a data availability statement. This statement should provide the following information, where applicable:

- Accession codes, unique identifiers, or web links for publicly available datasets
- A list of figures that have associated raw data
- A description of any restrictions on data availability

Raw sequencing data have been deposited in the European Genome-phenome Archive (EGA, www.ebi.ac.uk/ega/). Accession numbers are EGAD00001006574 (bulk mRNA-seq) and EGAD00001006296 (WGS & scRNA-seq). The data is available under restricted access. Access can be granted by contacting biobank@prinsesmaximacentrum.nl (EGAD00001006574) or datasharing@sanger.uk.ac (EGAD00001006296). DNA methylation data are available under GEO (www.ncbi.nlm.nih.gov/geo/) accession number GSE161814. Processed scRNA-seq data (derived from raw sequencing data deposited under accession number EGAD00001006296) are available at https://github.com/kheleon/mrt-paper52. Content includes filtered_gene_bc_matrices and filtered_feature_bc_matrix folders from cellranger output. The fetal mouse neural crest dataset18 was extracted from GEO (www.ncbi.nlm.nih.gov/geo/, accession number GSE129114). The second dataset of fetal mouse organogenesis19 was extracted from GEO (www.ncbi.nlm.nih.gov/geo/, accession number GSE119945). Gene expression data of the paediatric renal tumour biobank17 was extracted from EGA (www.ebi.ac.uk/ega/, accession number EGAD00001005318 and EGAD00001005319). Gene expression data of normal tissues was extracted from GTEx portal (https://gtexportal.org/home/datasets, GTEx_Analysis_2017-06-05_v8_RNASeQCv1.1.9_gene_median_tpm.gct.gz). The remaining data are available within the Article, Supplementary Information, Supplementary Data and Source Data provided with this paper or are available from the authors upon request.

Field-specific reporting

Please select the one below that is the best fit for your research. If you are not sure, read the appropriate sections before making your selection.

☒ Life sciences ☐ Behavioural & social sciences ☐ Ecological, evolutionary & environmental sciences

For a reference copy of the document with all sections, see nature.com/documents/nr-reporting-summary-flat.pdf

Life sciences study design

All studies must disclose on these points even when the disclosure is negative.

Sample size	For genetic lineage tracing experiments, we aimed to validate results in at least n=2 independent patient donors. Because of tumor rarity and complex requirements for tissue acquisition, we were unable to extent the study to more than 2 patients.<br>For in vitro experiments, we aimed to use n=3 independent patient-derived organoid lines to achieve adequate confidence of obtained results.
Data exclusions	A cervical spine sample from PD46555 was excluded from further analysis after pathological examination revealed abundant tumour infiltration (see Methods). Exclusion criteria were pre-established.
Replication	To verify the reproducibility of experimental findings, we performed independent replications of experiments. The n of independent experiments is indicated in the figure legends or in the Methods section under Statistics and reproducibility.
Randomization	Not relevant. There was no allocation of samples to experimental groups.
Blinding	All experiments, when relevant, were performed blinded. INI1 staining in Fig. 1 was assessed by a pathologist without prior knowledge of sample details.

Reporting for specific materials, systems and methods

We require information from authors about some types of materials, experimental systems and methods used in many studies. Here, indicate whether each material, system or method listed is relevant to your study. If you are not sure if a list item applies to your research, read the appropriate section before selecting a response.

Materials & experimental systems

n/a	Involved in the study	
	x	Antibodies
x		Eukaryotic cell lines
x		Palaeontology and archaeology
x		Animals and other organisms
	x	Human research participants
x		Clinical data
x		Dual use research of concern

Methods

n/a	Involved in the study	
x		ChIP-seq
x		Flow cytometry
x		MRI-based neuroimaging

Antibodies

Antibodies used	For immunohistochemical staining the following primary antibody was used:<br>- INI1 (BD Transduction Laboratories, 612111, 1:400).<br>For immunofluorescence the following dyes/antibodies were used:<br>-DAPI (Thermo Fisher, D9542, 1:1000)<br>-Alexa Fluor 647 Phalloidin (Thermo Fisher, A22287, 1:200)<br>-MMP2 (Thermo Fisher, MA5-13590, 1:500)<br>For western blot, the following primary antibodies were used:<br>- SMARCB1/INI1 (Santa Cruz Biotechnology, A-5, sc-166165, 1:1000)<br>- beta-tubulin (Santa Cruz Biotechnology, H235, sc-9104, 1:1000).
Validation	All antibodies used in this study were validated by the manufacturers. Additionally, on the manufacturer's antibody web page, references to other studies that made use of the antibody are provided.<br>https://www.bdbiosciences.com/us/reagents/research/antibodies-buffers/cell-biology-reagents/cell-biology-antibodies/purified-mouse-anti-baf47-25baf47/p/612111<br>https://www.thermofisher.com/order/catalog/product/D1306#/D1306<br>https://www.thermofisher.com/order/catalog/product/A22287#/A22287<br>https://www.thermofisher.com/antibody/product/MMP2-Antibody-clone-CA-4001-or-CA719E3C-Monoclonal/MA5-13590<br>https://www.scbt.com/p/ini1-antibody-a-5<br>https://www.scbt.com/p/beta-tubulin-antibody-h-235

Human research participants

Policy information about studies involving human research participants

Population characteristics	Material from patients who were diagnosed with MRT were included in this study. Covariates that were taken into account are age and sex, which were not expected to affect the obtained results.
Recruitment	All participants that signed informed consent were recruited in this study. Therefore, there are no self-selection biases that may have influenced the results.
Ethics oversight	Tissues were obtained as part of the SIOP2001 study approved by the medical ethical committees of the institutes involved (Ethical Committee University Leuven (Belgium), Medical ethical committee of the Erasmus Medical Centre Rotterdam (the Netherlands)), or from patients enrolled in the 'Investigating how childhood tumours and congenital disease develop' (NHS National Research Ethics Service reference 16/EE/0394) or through the UK IMPORT study (NHS National Research Ethics Service reference 12/LO/0101). Informed consent was provided by all participants.

Note that full information on the approval of the study protocol must also be provided in the manuscript.
